# Longitudinal Trends in Pediatric Survival by Congenital Heart Defect in Texas, 1999 to 2017

**DOI:** 10.1016/j.jacadv.2025.101812

**Published:** 2025-05-19

**Authors:** Sara B. Stephens, Shaine A. Morris, Renata H. Benjamin, Mark A. Canfield, Charles J. Shumate, Ruosha Li, Cecilia Cazaban-Ganduglia, A.J. Agopian

**Affiliations:** a Department of Epidemiology, School of Public Health, The University of Texas Health Science Center at Houston, Houston, Texas, USA; b Division of Pediatric Cardiology, Department of Pediatrics, Texas Children's Hospital and Baylor College of Medicine, Houston, Texas, USA; c Texas Department of State Health Services, Austin, Texas, USA; d Department of Biostatistics and Data Science, School of Public Health, UTHealth School of Public Health, Houston, Texas, USA; e Center for Healthcare Data, Department of Management, Policy, and Community Health, UTHealth School of Public Health, Houston, Texas, USA

**Keywords:** congenital heart disease, double outlet right ventricle, hypoplastic left heart syndrome, mortality, time trends, tricuspid atresia

## Abstract

**Background:**

Despite previously improved survival among children with congenital heart defects (CHDs), U.S. population-level evaluations of survival within recent years are scarce.

**Objectives:**

The purpose of this study was to describe the survival landscape among children with CHDs in a large population-based birth defects registry overall and by CHD lesion.

**Methods:**

This population-based cohort study evaluated 1999 to 2017 live births with ≥1 major CHD in the statewide Texas Birth Defects Registry. Variables included CHD lesion, demographics, gestational age at birth (term/preterm), low birthweight (<2,500 g at birth), among others. Kaplan-Meier analyses were used to describe survival to 7 days, 28 days, 1 year, 5 years, and 10 years of life. Kaplan-Meier survival estimates were generated for 1-year survival for CHDs overall by lesion, using log-rank tests assessing differences by exposure.

**Results:**

Of 61,656 children with CHDs, survival was 98.1% and 90.7% at 7 days and 10 years, respectively, and substantially varied by lesion (range, 50.0% to 97.3% 10-year survival). Survival longitudinally improved for complex lesions including hypoplastic left heart syndrome (48.7% 1-year survival for cases born 1999-2004 vs 64.8% in 2014-2017; *P* < 0.0001). One-year survival differed by maternal race/ethnicity (eg, 58.3% for cases with complex pulmonary atresia born to non-Hispanic Black mothers vs 80.5% for non-Hispanic White mothers, *P* = 0.01), sex, gestational age, birthweight, and extracardiac defect status.

**Conclusions:**

One-year survival improved for most CHDs over recent decades, although survival varies widely by CHD and characteristics. Findings have implications for clinical counseling, population-level resource and research planning, and reinforce the need for mitigation of disparities among individuals with CHDs.

Congenital heart defects (CHDs) are the most common birth defect type and confer markedly high morbidity, mortality, and health care expenditures.^1^, ^2^, ^3^, ^4^, ^5^, ^6^ Survival in patients with CHDs has markedly improved over time in part due to expanded use of fetal echocardiography for prenatal CHD diagnosis to plan for delivery, changes in optimal gestational age at delivery, refinements in cardiac surgical technique, and improvements in critical care.^7^, ^8^, ^9^, ^10^, ^11^

Within the last several years (eg, since 2013), comprehensive descriptions of lesion-specific survival across the full spectrum of CHDs among U.S. births are scarce. This gap yields an incomplete understanding of contemporaneous U.S.-based survival estimates among children with CHDs, particularly as treatment advances may impact survival trajectories. To address this, findings have been extrapolated from tertiary care center studies, which are not population-based, often focus only on postoperative mortality, and do not include patients without surgical intervention. Thus, there remains a paucity of recent U.S. studies identifying survival beyond the preoperative and postoperative periods among all children with CHDs, delineated by CHD type.

Such gaps have made it challenging to understand contemporaneous population-level survival trajectories. This information may be critical for identifying future health care needs for a population anticipated to have more adults surviving with CHDs in the future compared to past decades.^12^ We aimed to characterize survival in a population-based cohort of infants and children with CHDs using a large birth defect registry.

## Methods

### Study population and case data

The data source was the Texas Birth Defects Registry (TBDR), evaluating births recorded 1999 to 2017.^13^ The TBDR is managed by the Birth Defects Epidemiology and Surveillance Branch at the Texas Department of State Health Services (DSHS). Using active surveillance methods, the TBDR collects data on birth defects documented by age 1 year among all pregnancy outcomes delivered by mothers residing in Texas in hospitals, birthing centers, and relevant clinics, representing a population of >7 million live births statewide.^13^ TBDR staff have legislative authority to collect TBDR data on all deliveries within Texas without individual consent (Texas Health and Safety Code, Chapter 87; Texas Administrative Code, Title 25, Part 1, Chapter 37, Subchapter P, Rules 37.301-37.306).

Registry staff review medical records to abstract maternal and infant characteristics and assign a modified Centers for Disease Control and Prevention/British Pediatric Association (BPA) standard birth defect code for each infant birth defect diagnosis. Diagnoses are classified as “definite” or “possible or probable,” and only those considered to be “definite” were included in this study. Death records from the Vital Statistics Section at Texas DSHS' Center for Health Statistics and out-of-state death certificates from states with an interagency agreement with Texas are routinely linked to cases in the TBDR.^14^ The protocol for this study was approved by the Institutional Review Board at Texas DSHS and the Committee for the Protection of Human Subjects at University of Texas Health Science Center at Houston.

We included live-born individuals born ≥23 weeks' gestation with ≥1 major CHD codes (defined by the National Birth Defects Prevention Study or the TBDR) delivered between January 1, 1999, and December 31, 2017.^14^ CHD BPA codes were classified using CHD categories designated by the Core Cardiac Lesion Outcome Classification (C-CLOC) system as previously described.^15^ Briefly, the C-CLOC system was designed to categorize CHDs into mutually exclusive CHD categories (hierarchically grouped based on the most severe CHD code present) optimized for outcome-based association analyses in population-based registries.

### Outcome ascertainment

Using TBDR records, the primary outcome was 1-year survival, with secondary analyses for time points 7 days, 28 days (neonatal), 5 years, and 10 years of life. Children without a documented death identified on medical or linked state death records were presumed to be alive at censoring (December 31, 2018). Subjects with a death record without an available date of death were excluded (n = 123). For each time point, follow-up duration was calculated as either: 1) number of days alive at the end of the study period (for those surviving through December 31, 2018); or 2) the number of days alive until date of death for those that died before December 31, 2018.

### Additional variables

Additional variables of interest included birth year, maternal race/ethnicity (Hispanic, non-Hispanic [NH] White, NH Black, or NH additional groups), sex, gestational age at birth, low birthweight (LBW) status (<2,500 g at birth), and presence of concurrent genetic and extracardiac birth defects (3 groups).^16^ Birth year was evaluated as continuous and categorized variables (1999-2004, 2005-2009, 2010-2013, or 2014-2017). Maternal race/ethnicity was self-reported and ascertained via vital records. Gestational age at birth was categorized using a modified version of the World Health Organization's grouping (<32 weeks, 32 to <37 weeks, ≥37 weeks) based on weeks since the first day of the mother's last menstrual period.^17^ Cases were hierarchically stratified into: 1) genetic (ie, those with diagnosis associated with a documented genetic or environmental syndrome such as Marfan syndrome, 22q11.2 deletion syndrome, or fetal alcohol syndrome); 2) nongenetic with extracardiac defect; or 3) nongenetic without extracardiac defects.^16^ Extracardiac defects were defined as a definite diagnosis of a major defect outside of the cardiovascular system (BPA codes <745.000 or >747.490).

### Statistical analysis

All analyses were separately conducted for CHDs overall and, except when noted below, for each specific CHD lesion. Kaplan-Meier (KM) survival by age 7 days, 28 days, 1 year, 5 years, and 10 years stratified by infant and maternal characteristics were calculated with 95% CIs using birth as day 0 with subjects censored at age at last follow-up (capping at the maximum follow-up time for each analysis). For mortality by 7 days, 28 days, and 1 year, the frequency of death at each time point was plotted on a stacked bar chart by CHD type. Median follow-up time and IQR were reported.

KM curves were then generated to compare survival by 1 year by birth year group, maternal race/ethnicity, neonate sex, gestational age, LBW status, and genetic/extracardiac defect status. Log-rank test was used to evaluate differences in 1-year survival curves between characteristics. KM and log-rank analyses were then repeated, stratified by CHD category. KM survival estimates for mortality within the first year of life were plotted by birth year on bar charts with corresponding KM CIs among subjects with: 1) CHDs overall; and 2) critical CHDs. Critical CHDs were defined as aortic arch obstruction, dextro-transposition of the great arteries (TGA), double outlet right ventricle (DORV), Ebstein anomaly, hypoplastic left heart syndrome (HLHS), type B interrupted aortic arch, pulmonary atresia (any subtype), tetralogy of Fallot, total anomalous pulmonary venous return, tricuspid atresia, or truncus arteriosus, as consistent with prior literature.^18^ Joinpoint regression was used to assess changes in infant mortality over time for overall CHDs, with average annual percent change (AAPC) denoted for each group.^19^ As a subanalysis, we then repeated joinpoint analysis limited to cases with critical CHDs. Comparisons with n < 5 in a cell were not performed. Joinpoint regression was performed using Joinpoint Regression Program version 4.7.0.0. Remaining analyses were conducted on SAS software (Version 9.4, SAS Institute Inc).

## Results

**Figure 1:**
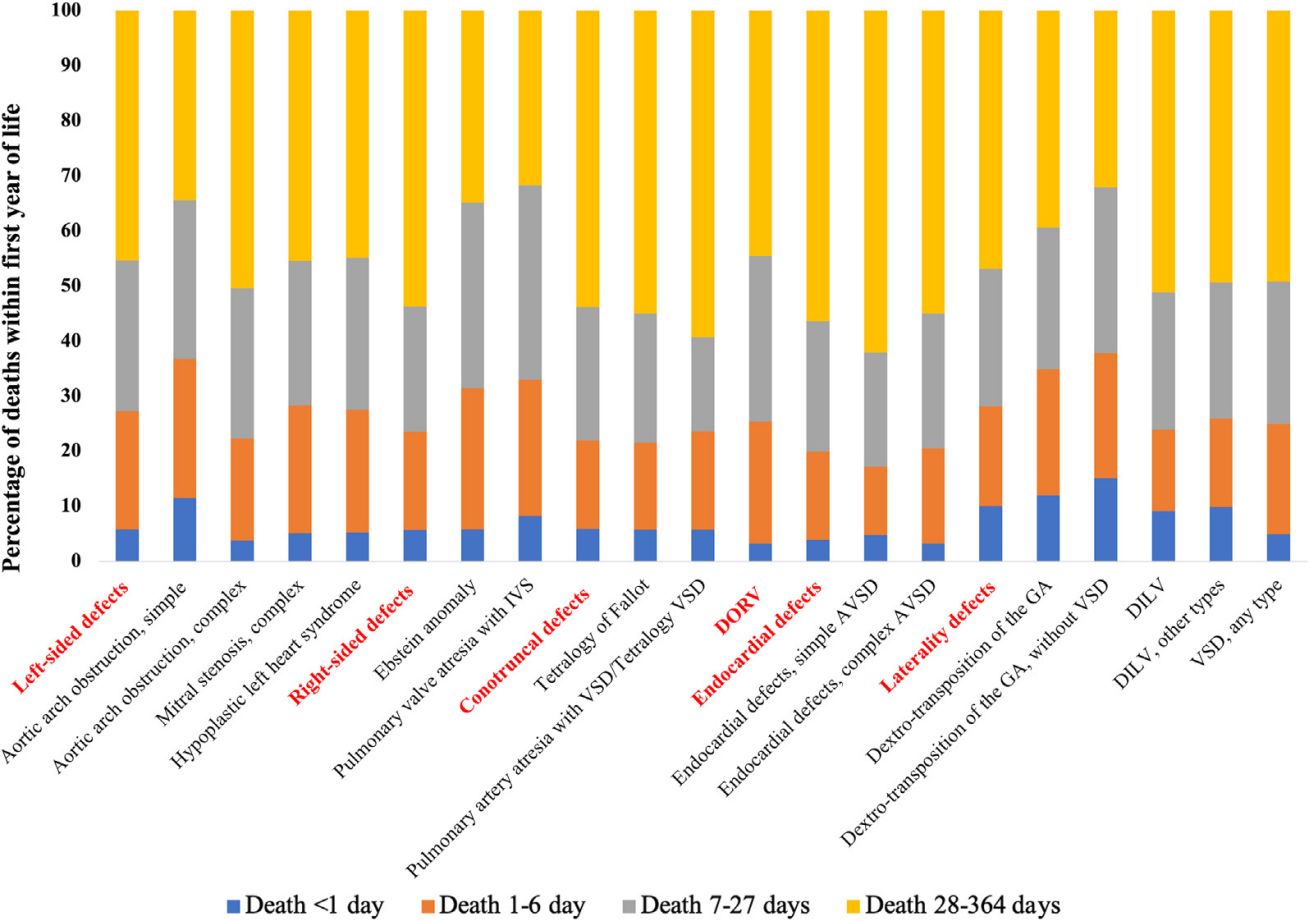
Percentage of Deaths Among Infants With Congenital Heart Defects, Texas, 1999 to 2017 Births AVSD = atrioventricular septal defect; DILV = double inlet left ventricle; DORV = double outlet left ventricle; GA = great arteries; IVS = intact ventricular septum; VSD = ventricular septal defect.

A total of 61,656 individuals born in 1999 to 2017 had an eligible CHD (Table 1). Among all infants with CHDs, the probability of survival decreased from 98.1% by age 7 days (95% CI: 98.0%-98.2%) to 90.7% by age 10 years (95% CI: 90.4%-90.9%; Central Illustration). Survival estimates varied by CHD subtype, with 11.7% of children with HLHS dying by 7 days compared to 0.2% of those with pulmonary stenosis. Children with HLHS had the lowest 10-year survival of all CHDs (50.0%). By 1 year, mortality most frequently occurred during 28 to 364 days of life for most CHDs, although mortality timing distribution varied by subtype (Figure 1, Supplemental Table 1).

**Table 1:** Median Follow-Up and Kaplan-Meier Survival Estimates by Congenital Heart Defect, 1999-2017 Births

	N	Follow-Up, y	7 Days (95% CI)	28 Days (95% CI)	1 Year (95% CI)	5 Years (95% CI)	10 Years (95% CI)
All CHDs	61,656	8.24 (3.78-13.03)	98.1 (98.0-98.2)	96.1 (95.9-96.2)	92.2 (92.0-92.4)	91.1 (90.9-91.3)	90.7 (90.4-90.9)
All left-sided lesions	8,252	6.52 (2.43-11.72)	95.7 (95.2-96.1)	91.4 (90.8-92.0)	84.2 (83.4-85.0)	81.9 (81.0-82.7)	81.4 (80.5-82.3)
Arch obstruction, simple	2,623	7.03 (3.41-11.65)	97.6 (96.9-98.1)	95.7 (94.8-96.4)	93.4 (92.3-94.3)	92.4 (91.3-93.3)	92.2 (91.1-93.2)
Arch obstruction, complex	2,454	6.98 (2.42-12.16)	96.6 (95.8-97.2)	92.4 (91.3-93.4)	84.6 (83.1-86.0)	82.2 (80.6-83.6)	81.7 (80.1-83.2)
Congenital aortic stenosis	722	8.96 (4.61-13.87)	98.2 (96.9-99.0)	96.4 (94.8-97.5)	93.2 (91.1-94.8)	92.5 (90.3-94.2)	92.5 (90.3-94.2)
Mitral stenosis, simple	502	6.64 (2.78-11.95)	97.0 (95.1-98.2)	95.2 (93.0-96.8)	91.6 (88.8-93.7)	89.5 (86.4-91.9)	89.5 (86.4-91.9)
Mitral stenosis, complex	425	5.62 (1.29-11.04)	93.4 (90.6-95.4)	87.3 (83.7-90.1)	76.7 (72.4-80.4)	74.9 (70.5-78.8)	73.6 (69.0-77.7)
Cor triatriatum	68	7.33 (3.49-13.11)	–a	–a	89.7 (79.6-95.0)	88.1 (77.7-93.9)	88.1 (77.7-93.9)
Supravalvar aortic stenosis	91	8.89 (4.56-14.80)	–a	–a	93.4 (85.9-97.0)	93.4 (85.9-97.0)	91.1 (81.6-95.9)
Hypoplastic left heart synd.	1,172	2.42 (0.09-8.19)	88.3 (86.3-90.0)	76.5 (74.0-78.9)	57.4 (54.5-60.2)	51.2 (48.3-54.1)	50.0 (47.0-52.9)
All right-sided lesions	5,689	8.29 (4.05-12.94)	98.5 (98.1-98.8)	97.0 (96.5-97.4)	93.5 (92.8-94.1)	92.6 (91.9-93.2)	92.0 (91.2-92.7)
Ebstein anomaly	420	6.66 (1.74-13.17)	93.6 (90.8-95.5)	86.9 (83.3-89.8)	79.8 (75.6-83.3)	78.8 (74.6-82.4)	77.9 (73.6-81.7)
Pulmonary stenosis	3,707	8.61 (4.77-12.99)	99.8 (99.6-99.9)	99.6 (99.4-99.8)	98.3 (97.8-98.6)	97.8 (97.3-98.2)	97.4 (96.8-97.9)
Pulmonary atresia, IVS	356	5.87 (1.06-12.41)	92.1 (88.8-94.5)	83.7 (79.4-87.2)	76.1 (71.3-80.0)	75.1 (70.3-79.3)	73.8 (68.7-78.1)
Tricuspid atresia, all	408	6.43 (2.05-12.50)	95.6 (93.1-97.2)	92.4 (89.4-94.6)	80.1 (75.9-83.7)	77.1 (72.6-80.9)	75.9 (71.3-79.9)
Tricuspid atresia, normal GA	309	6.34 (1.74-12.48)	95.8 (92.9-97.5)	91.9 (88.3-94.5)	79.3 (74.3-83.4)	76.4 (71.2-80.8)	75.8 (70.6-80.3)
Tricuspid atresia, malposed GA	98	6.47 (3.01-12.17)	98.0 (92.1-99.5)	93.9 (86.9-97.2)	82.7 (73.6-88.8)	79.0 (69.3-86.0)	75.8 (65.3-83.5)
All conotruncal defects	3,735	7.23 (2.52-12.77)	96.8 (96.2-97.3)	93.3 (92.4-94.0)	85.4 (84.2-86.4)	83.2 (81.9-84.3)	82.4 (81.1-83.6)
Tetralogy of Fallot	2,083	7.97 (3.39-13.26)	97.8 (97.1-98.4)	95.4 (94.5-96.3)	90.0 (88.5-91.1)	88.4 (86.9-89.7)	88.0 (86.5-89.3)
Pulmonary atresia w/VSD/TOF	567	5.57 (1.01-11.68)	94.2 (91.9-95.8)	89.9 (87.2-92.2)	75.3 (71.5-78.7)	70.0 (66.0-73.7)	68.9 (64.8-72.6)
Pulmonary atresia, complex	138	4.05 (0.23-10.05)	92.0 (86.1-95.5)	84.1 (76.8-89.2)	69.6 (61.1-76.5)	68.0 (59.5-75.1)	68.0 (59.5-75.1)
Truncus arteriosus	381	6.18 (0.63-11.80)	94.2 (91.4-96.2)	86.6 (82.8-89.7)	73.2 (68.5-77.4)	70.4 (65.6-74.8)	68.5 (63.5-73.0)
Truncus with IAA	37	2.98 (0.20-10.10)	–a	86.5 (70.5-94.1)	62.2 (44.6-75.6)	54.1 (36.9-68.4)	54.1 (36.9-68.4)
Truncus without IAA	344	6.54 (0.83-11.95)	94.2 (91.1-96.2)	86.6 (82.6-89.8)	74.4 (69.5-78.7)	72.2 (67.1-76.7)	70.1 (64.8-74.8)
Interrupted aortic arch, type B	217	4.86 (1.16-10.95)	96.3 (92.8-98.1)	87.6 (82.4-91.3)	75.6 (69.3-80.8)	73.6 (67.2-79.0)	72.7 (66.0-78.2)
Right arch	174	5.89 (2.38-11.34)	–a	97.1 (93.2-98.8)	94.8 (90.3-97.3)	93.9 (89.0-96.7)	92.8 (87.0-96.0)
Double aortic arch	253	7.49 (3.78-12.80)	–a	–a	96.8 (93.8-98.4)	96.3 (93.1-98.1)	96.3 (93.1-98.1)
DORV	1,116	5.28 (0.44-11.04)	92.9 (91.3-94.3)	84.5 (82.3-86.5)	72.0 (69.3-74.6)	69.0 (66.1-71.6)	67.5 (64.6-70.2)
DORV, TGA (Taussig-Bing)	135	9.05 (4.45-13.06)	–a	94.1 (88.5-97.0)	86.7 (79.7-91.4)	84.4 (77.0-89.5)	84.4 (77.0-89.5)
DORV, normal GA/TOF-type	433	6.61 (0.94-12.34)	93.8 (91.0-95.7)	85.2 (81.5-88.2)	74.6 (70.2-78.4)	73.2 (68.8-77.1)	71.5 (66.9-75.6)
DORV, complex	328	3.69 (0.41-9.23)	–a	83.2 (78.7-86.9)	69.8 (64.5-74.5)	65.4 (59.9-70.3)	63.1 (57.3-68.3)
DORV, mitral atresia	220	3.02 (0.11-8.85)	–a	79.1 (73.1-83.9)	61.4 (54.6-67.4)	56.2 (49.3-62.6)	54.8 (47.8-61.3)
All endocardial defects	2,906	6.59 (1.64-12.53)	95.9 (95.1-96.6)	91.0 (89.9-92.0)	79.4 (77.9-80.8)	75.7 (74.1-77.3)	74.6 (72.9-76.2)
Primum atrial septal defect	154	6.26 (2.39-11.01)	96.1 (91.5-98.2)	90.9 (85.1-94.5)	85.1 (78.4-89.8)	81.5 (74.3-86.8)	81.5 (74.3-86.8)
AV septal defect, simple	1,292	8.15 (3.07-13.60)	97.8 (96.8-98.4)	95.0 (93.7-96.1)	86.9 (85.0-88.6)	84.8 (82.7-86.7)	83.7 (81.5-85.7)
AV septal defect, complex	1,429	5.10 (0.55-11.31)	94.2 (92.8-95.3)	87.5 (85.7-88.9)	71.7 (69.2-73.9)	66.5 (64.0-68.9)	65.3 (62.7-67.7)
All laterality defects	2,018	8.23 (3.13-13.62)	95.7 (94.7-96.5)	91.9 (90.6-93.0)	84.7 (83.1-86.2)	82.7 (81.0-84.3)	82.2 (80.4-83.8)
Congenitally corrected TGA	157	8.82 (3.51-14.12)	95.5 (90.9-97.8)	93.0 (87.7-96.1)	86.0 (79.5-90.5)	84.6 (77.8-89.4)	84.6 (77.8-89.4)
dTGA	1,255	9.15 (4.33-13.90)	97.0 (95.9-97.8)	94.7 (93.4-95.8)	91.3 (89.6-92.7)	90.5 (88.8-92.0)	90.4 (88.6-91.9)
dTGA with VSD	503	9.60 (4.78-13.74)	97.6 (95.8-98.6)	95.4 (93.2-96.9)	90.9 (88.0-93.1)	89.6 (86.6-92.0)	89.3 (86.2-91.7)
dTGA without VSD	632	9.47 (4.26-14.44)	96.8 (95.1-97.9)	94.3 (92.2-95.9)	91.6 (89.2-93.5)	91.1 (88.6-93.1)	91.1 (88.6-93.1)
DILV	597	5.91 (0.42-12.42)	93.0 (90.6-94.8)	85.6 (82.5-88.2)	70.5 (66.7-74.0)	65.9 (61.9-69.6)	64.4 (60.3-68.1)
DILV, L-looping	97	8.15 (3.92-12.51)	100 (-)	–a	85.6 (76.8-91.2)	83.5 (74.5-89.5)	83.5 (74.5-89.5)
DILV, other	500	5.13 (0.27-12.38)	91.6 (88.8-93.7)	83.6 (80.1-86.6)	67.6 (63.3-71.5)	62.5 (58.0-66.6)	60.7 (56.1-64.9)
Other defect types							
Aortopulmonary window	45	8.22 (4.42-10.83)	–a	88.9 (75.3-95.2)	86.7 (72.7-93.8)	84.4 (70.0-92.2)	84.4 (70.1-92.2)
Partial anomalous PV return	165	6.83 (3.18-11.33)	96.4 (92.1-98.3)	94.6 (89.8-97.1)	87.9 (81.8-92.0)	87.3 (81.2-91.5)	86.4 (80.1-90.9)
Total anomalous PV return	626	8.57 (3.05-13.43)	96.5 (94.7-97.7)	93.1 (90.9-94.9)	86.3 (83.3-88.7)	85.9 (82.9-88.4)	85.7 (82.7-88.2)
VSD	36,966	8.84 (4.47-13.37)	99.2 (99.1-99.3)	98.3 (98.2-98.5)	96.7 (96.5-96.9)	96.2 (96.0-96.4)	96.0 (95.8-96.2)
VSD, muscular	7,644	3.17 (2.03-4.31)	99.8 (99.6-99.8)	99.3 (99.0-99.4)	98.2 (97.9-98.5)	97.7 (97.2-98.0)	97.3 (96.7-97.9)
VSD, membranous	1,935	3.12 (1.98-4.32)	99.0 (98.4-99.3)	97.8 (97.1-98.4)	95.0 (93.9-95.9)	94.6 (93.5-95.6)	94.6 (93.5-95.6)
VSD, other	27,382	11.1 (7.87-14.77)	99.0 (98.9-99.2)	98.1 (98.0-98.3)	96.4 (96.2-96.7)	95.9 (95.7-96.2)	95.7 (95.4-95.9)

AV = atrioventricular; CHD = congenital heart defect; dTGA = dextro-transposition of the great arteries; DILV = double inlet left ventricle; DORV = double outlet left ventricle; GA = great arteries; IAA = interrupted aortic arch; IVS = intact ventricular septum; PV = pulmonary venous; Synd. = syndrome; TGA = transposition of the great arteries; TOF = tetralogy of Fallot; Truncus = truncus arteriosus; VSD = ventricular septal defect.

a Survival estimates for CHDs with <5 deaths within a given category were not computed.

**Central Illustration:**
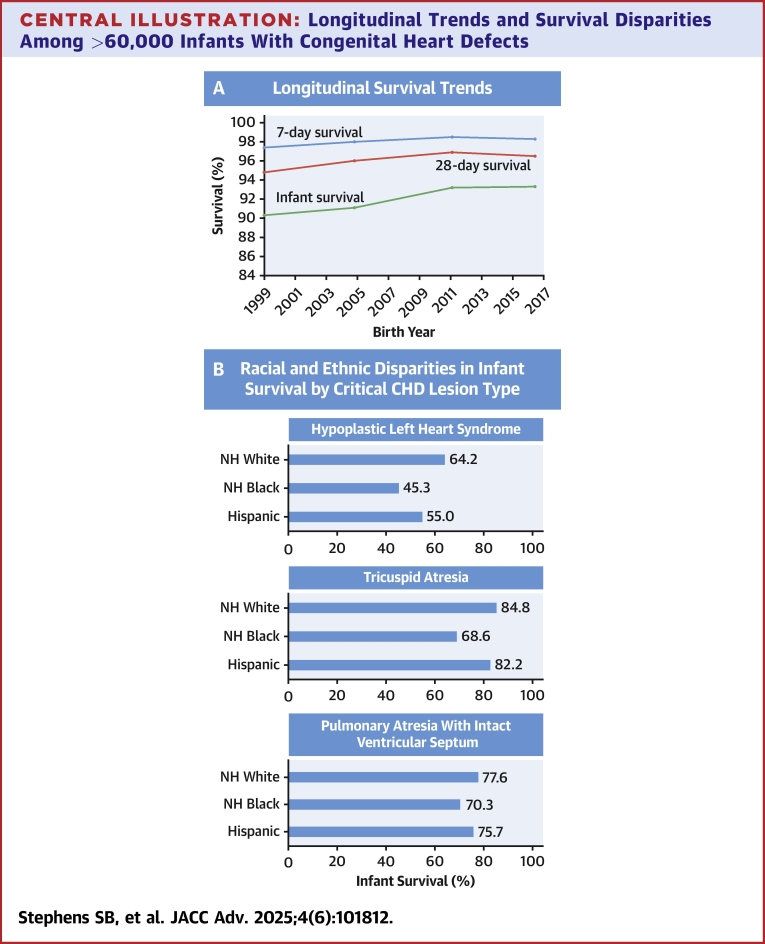
Longitudinal Trends and Survival Disparities Among >60,000 Infants With Congenital Heart Defects (A) Overall survival in children with CHD has markedly increased over the last 2 decades, with the largest improvements observed for infant survival. (B) While infant survival varied by CHD lesion type and was highest among those with critical CHDs, racial and ethnic disparities in infant survival persisted with the lowest survival often observed in non-Hispanic Black and Hispanic infants. CHD = congenital heart defect; other abbreviation as in Figure 3.

### One-year survival by birth era

**Figure 2:**
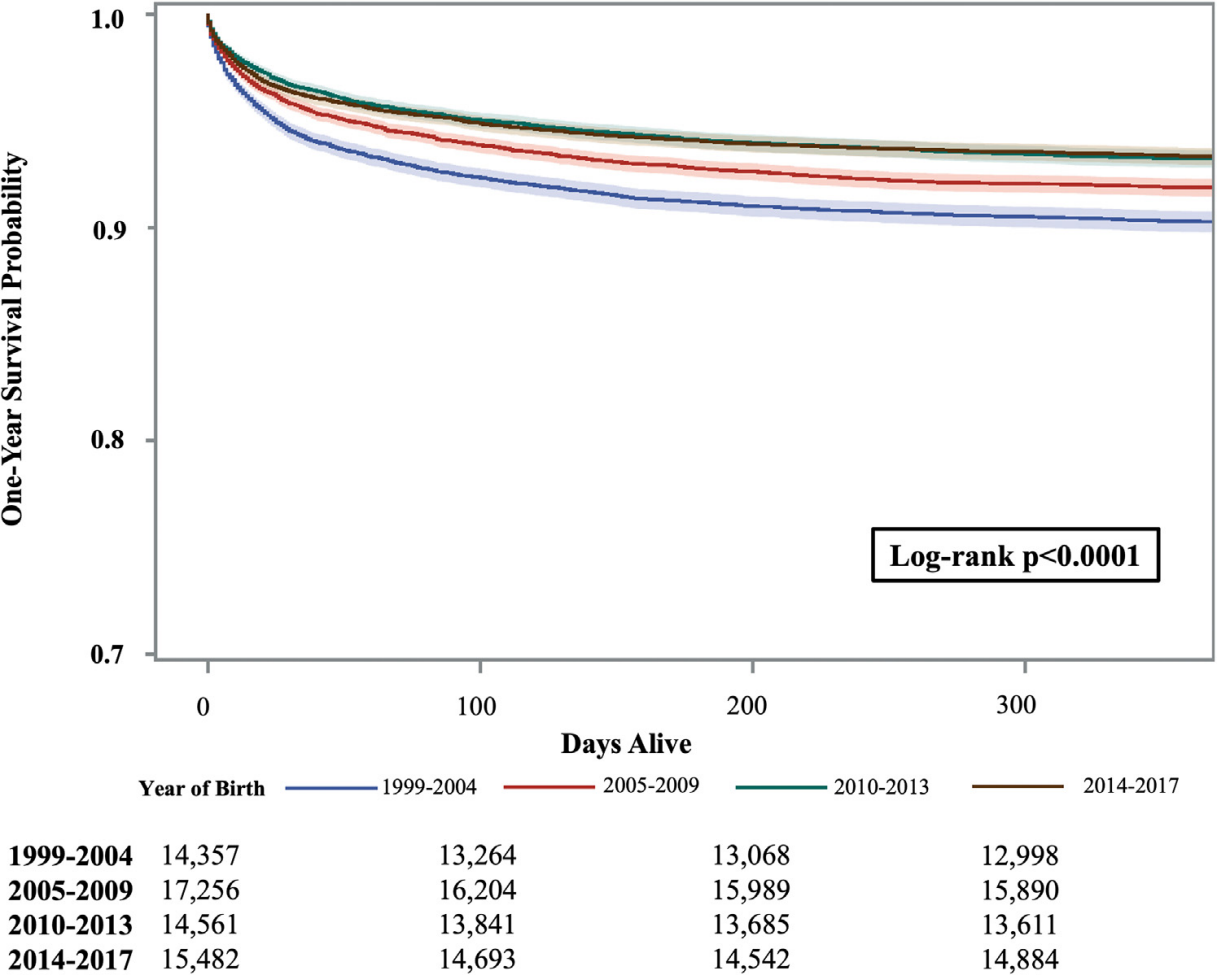
Kaplan-Meier Survival Estimates Among Infants With Congenital Heart Defect by Birth Year, Texas, 1999 to 2017 Births

Among all infants with CHDs, survival to age 1 year significantly improved over time (90.3% for 1999-2004 births vs 93.3% for 2014-2017 births; *P* < 0.0001) (Figure 2, Supplemental Table 2). In fact, a linear increase in 1-year survival was observed among all CHDs in the final joinpoint model, denoting a steady increase in 1-year survival until 2011 (AAPC of +0.4% from 1999 to 2010) with no significant change after (Supplemental Figure 1). Among infants with critical CHDs, a linear increase in 1-year survival was noted from 1999 to 2012, with an AAPC of +0.9% on joinpoint regression, after which time there was no significant change in 1-year survival (Supplemental Figure 2). Observed improvement in survival by era was primarily driven by improvements in complex lesions, such as aortic arch obstruction, HLHS, and pulmonary atresia with ventricular septal defect (VSD).

When evaluating by CHD subtype, survival significantly improved over time for 11 out of 39 (28.2%) CHDs assessed, most of which were complex CHDs (Supplemental Figure 3, Supplemental Table 2). The largest improvements in 1-year survival were observed among infants with pulmonary atresia with VSD (67.1% for 1999-2004 vs 84.5% for 2014-2017; *P* = 0.009) and those with DORV with mitral atresia (50.0% for 1999-2004 births vs 71.7% for 2014-2017 births; *P* = 0.13), although the latter was not statistically significant.

### 1-year survival by maternal race/ethnicity

**Figure 3:**
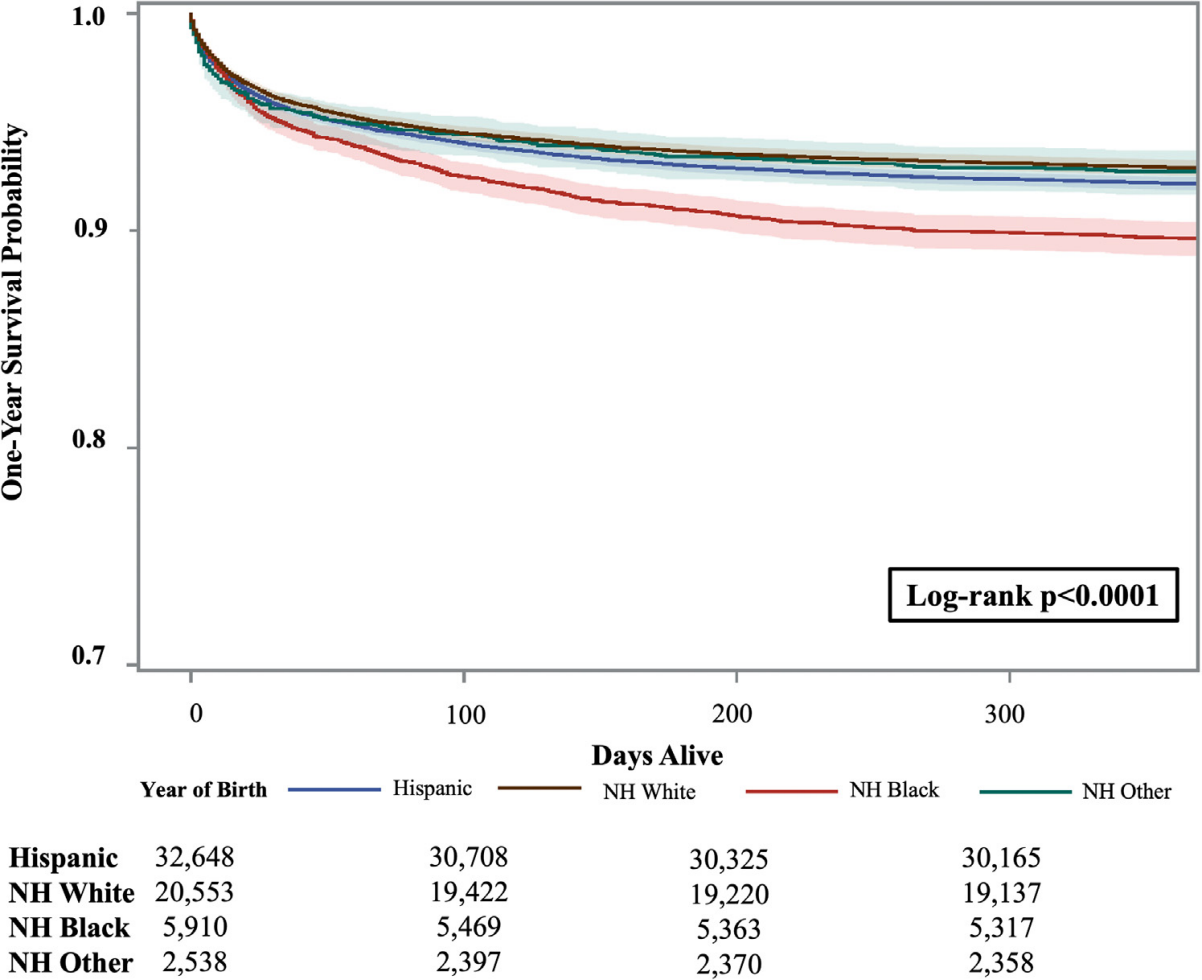
Kaplan-Meier Survival Estimates Among Infants With Congenital Heart Defect by Maternal Race/Ethnicity, Texas, 1999 to 2017 Births NH = non-Hispanic.

One-year survival for all cases with CHDs significantly differed by maternal race/ethnicity, with 92.9% of infants born to NH White mothers surviving to age 1 year compared to 92.2% and 89.7% of their counterparts born to Hispanic and NH Black mothers, respectively (*P* < 0.001) (Figure 3, Supplemental Figure 4, Supplemental Table 3). Survival significantly differed by maternal race/ethnicity for 9/24 CHDs assessed (37.5%) with the largest difference among those with complex pulmonary atresia in which 58.3%, 69.2%, and 80.5% of infants survived to age 1 year respectively for those born to NH Black, Hispanic, and NH White mothers (*P* = 0.010).

### One-year survival by infant sex

**Figure 4:**
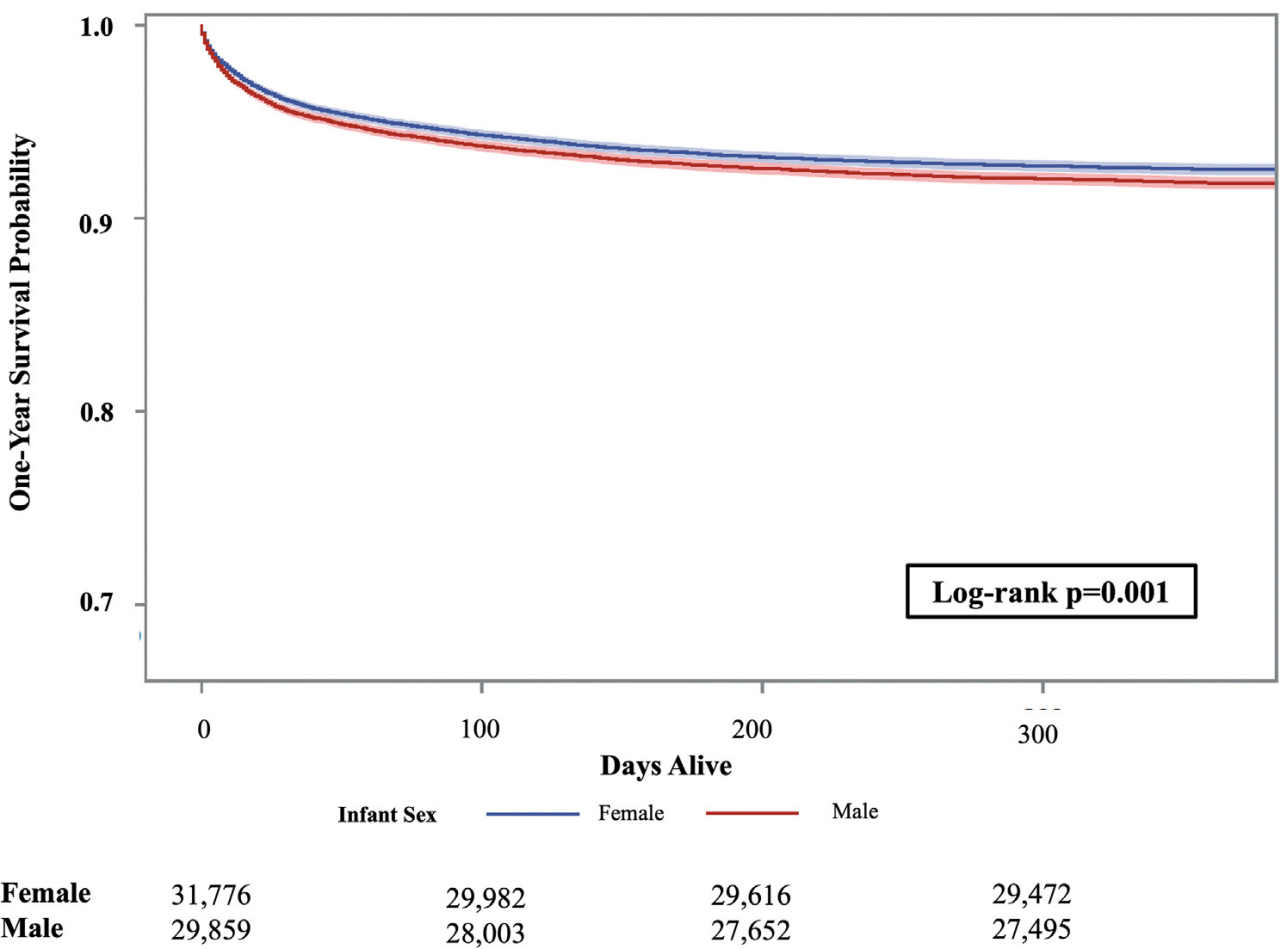
Kaplan-Meier Survival Estimates Among Infants With Congenital Heart Defect by Infant Sex, Texas, 1999 to 2017 Births

Survival to age 1 year among males with CHDs was significantly lower than females (*P* < 0.0001) (Figure 4, Supplemental Figure 5, Supplemental Table 4), although this was mostly driven by differential mortality in those with VSDs given the large number of cases (96.5% for males vs 96.9% females; *P* = 0.046). Notably, large statistically significant differences in 1-year survival by sex were observed for several complex lesions, including HLHS (61.3% for males vs 50.5% for females; *P* = 0.0002), DORV with TGA (89.9% vs 77.8%; *P* = 0.044), complex DORV (74.4% vs 63.7%; *P* = 0.040), congenitally corrected TGA (93.1% vs 75.9%; *P* = 0.0006), and other types of double inlet left ventricle (DILV) (72.7% vs 60.0%; *P* = 0.005).

### One-year survival by gestational age at birth

As expected, compared to those born at ≥37 weeks' gestation, infants with CHDs overall born at <32 weeks or between 32 and <37 weeks had significantly lower survival to 1 year (*P* < 0.0001) (Supplemental Figures 6 and 7, Supplemental Table 5). These differences were most notable among single ventricle lesions such as DILV, with 29.4% of cases with DILV born <32 weeks surviving to age 1 year compared to 53.3% of those born between 32 and <37 weeks, and 75.2% of those born ≥37 weeks (*P* < 0.0001) (Supplemental Table 5).

### One-year survival by low birthweight status

Among all infants with CHDs, survival to age 1 year was significantly lower among infants with LBW compared to those without (82.4% vs 95.3%; *P* < 0.0001) (Supplemental Figures 8 and 9, Supplemental Table 6). Similar to gestational age-stratified analyses, the largest differences in survival by LBW were observed among those with complex CHDs including HLHS (33.8% vs 62.7%; *P* < 0.0001), complex mitral stenosis (54.4% vs 86.0%; *P* < 0.0001), and complex DORV (48.3% vs 77.4%; *P* < 0.0001).

### 1-year survival by concurrent genetic diagnosis and extracardiac defects

Among all CHDs, children with a genetic diagnosis had significantly lower survival to age 1 year compared to those without a genetic diagnosis, regardless of presence of extracardiac defect status (*P* < 0.0001) (Supplemental Figures 10 and 11, Supplemental Table 7). When stratifying by CHD subtype, however, this difference in survival to 1 year was primarily noted between children with a genetic diagnosis and those with neither a genetic diagnosis nor an extracardiac defect based on their lack of overlapping CIs. This magnitude of difference, however, varied by subtype.

## Discussion

This is one of the first U.S. population-based studies to describe lesion-specific survival proportions across a comprehensive spectrum of CHDs that includes infants born after 2013. Our results highlight CHD lesion-specific and characteristic-specific differences in survival, with approximately 93% to 94% of children with CHDs born in 2010 to 2017 surviving to age 10 years. Over the 19-year study period, survival in infants with CHDs overall and critical CHDs overall steadily improved, with pronounced improvements for complex lesions including DORV with mitral atresia and pulmonary atresia with VSD. In HLHS, a CHD that is 100% lethal without surgical palliation, 1- year survival improved from 1999-2004 (49%) to 2010-2013 (65%; results including both those with and without surgical palliation for comprehensiveness). These improvements are remarkable in light of those reported in prior Texas estimates among 1995-1997 births reporting 1-year survival of 21% to 75%.^20^ Overall, results support the concept that survival has continued to increase in the US since the 1950s, which has since plateaued in recent years.^9^^,^^21^ Such findings must be considered in light of potential changes in prenatal detection or trends in termination of pregnancies over time, which this study did not account for.

Direct comparison of results to recent literature is difficult due to the lack of comparable, contemporary U.S. estimates, as U.S. registry studies have focused on birth years in prior decades and the small number of registry studies outside of the United States may not be generalizable due to differences in health care systems. With this in mind, the improvements in early survival among those with critical CHDs observed in this study are consistent with prior population-based studies outside of the United States.^9^^,^^22^, ^23^, ^24^, ^25^ Additionally, similar to population-based investigations of children with other birth defects, U.S. states, and study periods, racial and ethnic differences in 1-year survival among children with CHDs persist, with lower survival among infants born to NH Black and Hispanic women compared to that of NH White women.^5^^,^^26^, ^27^, ^28^, ^29^, ^30^ Noting that race/ethnicity represents a social construct rather than a biologic marker, differences in survival are likely driven by a complex array of factors influencing health and outcome differences at the individual-, institutional-, and population-levels including social determinants of health, clinical factors, and more nuanced confounders such as differential distribution of preterm birth by race/ ethnicity.^5^^,^^31^, ^32^, ^33^, ^34^ Investigators recognize the need to transition from describing disparities to future work: 1) identifying causal mechanisms underlying health outcome differences in CHDs; and 2) working toward developing targeted public health strategies aimed to improving outcomes among children with CHDs from historically marginalized populations. Results from this population-based study may elucidate which CHDs may be most amenable to disparity reduction.

Furthermore, male infants had lower survival to age 1 year compared to female infants for CHDs overall, consistent with that of prior literature.^33^^,^^35^ However, both magnitude and direction of sex-based differences varied by CHD subtype, with improved survival for females among those with VSDs and higher survival for males in complex lesions such as HLHS and complex DORV. Observed differences may be related to differences in patients undergoing CHD surgery and high-risk procedures, those with complex CHDs, or unrecognized sex-based genetic disorders like Turner syndrome and Kabuki syndrome type 2.^36^, ^37^, ^38^ Additional evaluation of hormonal, genetic, and behavioral variables is warranted to better elucidate mechanisms of sex-based differences in outcomes among children with CHDs.

Additionally, we observed lower survival among cases with CHDs born prematurely or with LBW for nearly every CHD evaluated. Neonates with CHDs are more frequently small for gestational age and have smaller fetal head circumference, total brain volume and intracranial cavity volume adjusted for gestational age, and more frequently abnormal extracardiac development.^39^, ^40^, ^41^, ^42^ Children with CHDs are more frequently spontaneously born prematurely, whereby preterm birth alone is a leading cause of morbidity and mortality in early life.^43^ Gestational age and birthweight are implicated in surgical decision-making and timing, as cardiac surgery may be associated with excess risk or infeasible for children with CHDs born small for gestational age or prematurely and subsequently may not be offered.^43^, ^44^, ^45^ Recognizing the profoundly worse outcomes among those with CHDs born prematurely, clinicians often delay delivery for as long as safely possible, ideally to 39 to 40 weeks' gestation, to improve neonatal outcomes.^46^^,^^47^ In response to this, the proportion of children with CHDs delivered prematurely has decreased, suggesting that preterm delivery in neonates with CHDs may be somewhat modifiable.^48^ Given the well-documented higher risk associated with characteristics evaluated, further population-based research is needed to elucidate the underlying mechanisms by which these characteristics impact outcomes among infants and children with CHDs, particularly as it relates to different racial ethnic groups and its association with modifiable social determinants of health.

Higher mortality was noted in nonsyndromic infants with extracardiac defects. However, it is well-known that infants with different genetic conditions and/or extracardiac defects demonstrate variable mortality.^38^^,^^49^ To generate optimal prognostic information, future work should examine these specific conditions in greater detail, especially as the proportion of children with CHDs with identified genetic conditions increases over time.^50^ While our descriptive framework helps to define the mortality burden at the population level and among key subgroups, analytic epidemiologic investigations will be useful to identify which clinical and socioeconomic determinants and combinations drive these survival differences.

A key strength of this study was the population-based data source, enabling inclusion of nearly all livebirths with CHDs in Texas during the study period and mitigating selection bias. Additionally, use of TBDR ensured large sample sizes of rarer CHDs. Survival estimates generated by C-CLOC were consistent with clinical expertise, suggesting that C-CLOC may be a beneficial approach for generating clinically relevant findings using population-based birth defects registry data in outcome studies.

### Study Limitations

Analyses were limited by lack of disease severity data within a given CHD. Data were ascertained from abstracted records, which may be susceptible to errors. Surgical data were not systematically collected or considered, which future studies need to consider.

## Conclusions

This comprehensive evaluation of survival among children with CHDs in Texas identified variable survival across a wide spectrum of CHDs, many of which have improved with clinical and surgical advancements. Despite this, survival differences by factors including race and ethnicity remain.. Findings may inform clinical prognostic counseling, research prioritization, and planning for health care and service resource allocation.

Perspectives **COMPETENCY IN MEDICAL KNOWLEDGE:** Among >60,000 children with CHD, approximately 92% of cases survived to 1 year and 90% survived to 10 years with variable survival by lesion. Using a novel CHD classification system informed by practicing clinicians, findings denote population-based estimates both within the contemporaneous era, and importantly, by CHD lesion. Despite overall survival improving from 1999 to 2018 for most lesions, NH Black and Hispanic infants for several CHD lesions had significantly lower survival compared to NH White infants. While these disparities are previously reported, lesion-specific survival estimates supply the literature with estimates that are both granular and generalizable.

**COMPETENCY IN SYSTEMS-BASED PRACTICE:** Given the substantial variation in survival by CHD type and paucity of population-based survival data by lesion, this study reports lesion-specific survival estimates that account for clinical modifiers using 19 years' worth of data. These results may improve personalized risk stratification for evidence-based care and guided decision-making for children with CHD, particularly within the first year of life.

**TRANSLATIONAL OUTLOOK 1:** Findings from this population-based study can help update CHD lesion-specific risk profiles, aiding cardiac providers in clinical counseling and prognostication.

**TRANSLATIONAL OUTLOOK 2:** Despite public health efforts in reducing disparities, disparities among children with CHD persist. Using evidence-based care, practitioners caring for children with CHD should continue working on a clinical basis to mitigate these differences.

## Funding support and author disclosures

This project was supported by a grant from the 10.13039/100009633Eunice Kennedy Shriver National Institute of Child Health & Human Development (R01 HD093660) and the 10.13039/100000030Centers for Disease Control and Prevention (CDC). Support for data collection was provided in part by the Maternal and Child Health Section, Texas Department of State Health Services, using Title V Maternal and Child Health Block Grant funds. This study was funded in part by a 10.13039/100000030CDC birth defects surveillance cooperative agreement with the Texas Department of State Health Services (HHS 00096260001) and 10.13039/100000102Health Resources and Services Administration (HRSA) Block Grant funds. The contents are those of the author(s) and do not necessarily represent the official views of, nor an endorsement, by HRSA or CDC. The authors have reported that they have no relationships relevant to the contents of this paper to disclose.
