# Supplementary material for: Longitudinal Trends in Pediatric Survival by Congenital Heart Defect in Texas, 1999 to 2017
Source: JACC Adv. 2025 May 19;4(6):101812. doi: 10.1016/j.jacadv.2025.101812 (PMC12149392; doi:10.1016/j.jacadv.2025.101812)
Supplement: Supplementary data [file mmc1.docx]

**SUPPLEMENTAL TABLE 1. Survival by Select Characteristics Among Infants with CHD, Texas, 1999-2017 Births**

| **Characteristic** | **Total N** | **7 D**  **(95% CI)** | **28 D**  **(95% CI)** | **1 Y**  **(95% CI)** | **5 Y**  **(95% CI)** | **10 Y**  **(95% CI)** |
| --- | --- | --- | --- | --- | --- | --- |
| 1999-2004 | 14,357 | 97.4 (91.7-97.6) | 94.8 (94.4-95.2) | 90.3 (89.8-90.7) | 89.0 (88.5-89.5) | 88.5 (88.0-89.0) |
| 2005-2009 | 17,256 | 98.0 (97.8-98.2) | 96.0 (95.7-96.3) | 91.9 (91.5-92.3) | 90.8 (90.4-91.3) | 90.5 (90.1-90.9) |
| 2010-2013 | 14,561 | 98.5 (98.3-98.7) | 96.9 (96.6-97.1) | 93.2 (92.8-93.6) | 92.2 (91.7-92.6) | 91.7 (91.1-92.2) |
| 2014-2017 | 15,482 | 98.3 (98.1-98.5) | 96.5 (96.2-96.8) | 93.3 (92.9-93.7) | 90.4 (87.4-92.6) | 90.4 (87.4-92.6) |
| Hispanic | 32,648 | 98.0 (97.8-98.1) | 96.0 (95.8-96.2) | 92.2 (91.9-92.4) | 91.1 (90.8-91.4) | 90.7 (90.3-91.0) |
| Non-Hispanic White | 20,553 | 98.3 (98.1-98.4) | 96.3 (96.1-96.6) | 92.9 (92.5-93.2) | 91.9 (91.5-92.2) | 91.5 (91.1-91.9) |
| Non-Hispanic Black | 5,910 | 98.1 (97.7-98.4) | 95.3 (94.7-95.8) | 89.6 (88.8-90.4) | 88.1 (87.3-88.9) | 87.4 (86.5-88.2) |
| Additional groups | 2,538 | 97.6 (96.9-98.1) | 95.8 (94.9-96.5) | 92.7 (91.6-93.7) | 91.6 (90.5-92.7) | 91.4 (90.2-92.5) |
| Male | 29,859 | 97.9 (97.7-98.1) | 95.8 (95.5-96.0) | 91.8 (91.5-92.1) | 90.6 (90.2-90.9) | 90.2 (89.8-90.5) |
| Female | 31,776 | 98.2 (98.1-98.4) | 96.3 (96.1-96.5) | 92.5 (92.2-92.8) | 91.6 (91.3-91.9) | 91.2 (90.8-91.5) |
| Born <32 weeks’ gestation | 4,479 | 93.5 (93.2-93.8) | 90.6 (90.2-90.9) | 86.3 (85.8-86.7) | 79.3 (78.0-80.4) | 78.8 (77.6-80.0) |
| Born 32 to <37 weeks’ gest.  Born >37 weeks’ gest. | 10,942  46,235 | 96.5 (96.3-96.6)  99.3 (99.3-99.3) | 95.4 (95.2-95.5)  98.9 (98.8-98.9) | 93.3 (93.1-93.5)  97.9 (97.8-97.9) | 86.8 (86.2-87.4)  93.2 (93.0-93.5) | 86.3 (85.6-86.9)  92.9 (92.6-93.1) |
| LBW | 14,212 | 94.7 (94.3-95.0) | 90.0 (89.5-90.5) | 82.3 (81.7-82.9) | 80.4 (79.8-81.1) | 79.8 (79.1-80.5) |
| No LBW | 47,302 | 99.1 (99.0-99.2) | 97.9 (97.8-98.1) | 95.3 (95.1-95.5) | 94.4 (94.2-94.6) | 94.0 (93.8-94.3) |
| Genetic | 10,332 | 94.7 (94.3-95.0) | 90.0 (89.5-90.5) | 82.4 (81.7-83.0) | 80.4 (79.8-81.1) | 79.8 (79.1-80.5) |
| Non-genetic + ECD | 7,774 | 96.5 (96.1-96.9) | 93.0 (92.4-93.5) | 86.1 (85.3-86.8) | 84.2 (83.3-85.0) | 83.5 (82.6-84.3) |
| Non-genetic - ECD | 43,550 | 99.3 (99.3-99.4) | 98.5 (98.3-98.6) | 97.0 (96.8-97.1) | 96.4 (96.3-96.6) | 96.2 (96.1-96.4) |

CHD: Congenital heart defects; CI: Confidence interval; D: Days; ECD: Extracardiac defects; Gest.: Gestation; LBW: Low birthweight; Y: Year.

**SUPPLEMENTAL TABLE 2. Survival to Age 1 Year by Birth Year Among Infants with CHD, Texas, 1999-2017 Births^a^**

| **Defect** | **1999-2004** | **2005-2009** | **2010-2013** | **2014-2017** | **p-value^a^** |
| --- | --- | --- | --- | --- | --- |
| All CHDs | 90.3 (89.8-90.7) | 91.9 (91.5-92.3) | 93.2 (92.8-93.6) | 93.3 (92.9-93.7) | **<0.0001** |
| All left-sided lesions | 79.1 (77.1-80.9) | 82.9 (81.3-84.4) | 86.2 (84.6-87.6) | 87.7 (86.2-88.9) | **<0.0001** |
| Arch obstruction, simple | 89.7 (86.6-92.2) | 93.0 (90.8-94.7) | 94.1 (92.0-95.6) | 95.2 (93.5-96.5) | **0.0014** |
| Arch obstruction, complex | 82.3 (78.8-85.3) | 84.9 (81.3-84.4) | 86.2 (84.6-87.6) | 87.7 (86.2-88.9) | 0.293 |
| Congenital aortic stenosis | 91.2 (86.3-94.5) | 92.0 (87.3-95.0) | 95.0 (90.6-97.4) | 94.7 (89.6-97.3) | 0.293 |
| Mitral stenosis, simple | 87.1 (78.4-92.5) | 90.1 (83.2-94.2) | 95.3 (89.8-97.8) | 92.5 (87.2-95.7) | 0.153 |
| Mitral stenosis, complex | 73.8 (62.6-82.0) | 75.8 (67.1-82.5) | 77.8 (68.7-84.5) | 78.6 (70.0-85.0) | 0.881 |
| Cor triatriatum | -- ^b^ | -- ^b^ | -- ^b^ | -- ^b^ | -- ^b^ |
| Supravalvar aortic stenosis | -- ^b^ | -- ^b^ | -- ^b^ | -- ^b^ | -- ^b^ |
| Hypoplastic left heart synd. | 48.7 (43.0-54.1) | 52.3 (46.5-57.7) | 64.7 (58.7-70.1) | 64.8 (59.0-70.0) | **<0.0001** |
| All right-sided lesions | 91.0 (89.3-92.3) | 93.3 (92.0-94.5) | 95.7 (94.5-96.7) | 93.7 (92.3-94.8) | **<0.0001** |
| Ebstein anomaly | 75.6 (67.1-82.2) | 77.7 (67.8-84.8) | 86.5 (78.3-91.8) | 79.4 (69.9-86.2) | 0.184 |
| Pulmonary stenosis | 98.0 (96.7-98.8) | 98.4 (97.4-99.0) | 98.7 (97.7-99.2) | 97.9 (96.8-98.7) | 0.579 |
| Pulmonary atresia, IVS | 63.0 (57.2-68.3) | 74.1 (68.7-78.8) | 78.2 (72.0-83.2) | 79.6 (74.2-83.9) | 0.515 |
| Tricuspid atresia | 70.8 (60.6-78.8) | 75.8 (66.1-83.0) | 82.5 (69.8-90.2) | 77.4 (68.2-84.2) | 0.747 |
| Tricuspid atresia, normal GA | 76.8 (67.1-83.9) | 80.0 (71.9-86.0) | 82.2 (72.6-88.7) | 81.9 (72.5-88.3) | 0.726 |
| Tricuspid atresia, malposed GA | 75.3 (64.1-83.5) | 79.3 (69.6-86.3) | 80.0 (68.6-87.6) | 82.9 (71.8-89.9) | 0.830 |
| All conotruncal defects | 80.3 (77.6-82.6) | 86.9 (84.7-88.9) | 86.3 (83.7-88.5) | 88.2 (86.1-90.1) | **<0.0001** |
| Tetralogy of Fallot | 88.0 (84.9-90.5) | 91.0 (88.3-93.1) | 90.3 (87.2-92.7) | 90.4 (87.6-92.6) | 0.423 |
| Pulmonary atresia w/ VSD/TOF | 67.1 (59.3-73.7) | 75.4 (68.2-81.3) | 78.0 (69.6-84.4) | 84.5 (76.5-89.9) | **0.0086** |
| Pulmonary atresia, complex | 71.9 (52.9-84.3) | 60.7 (40.4-76.0) | 71.0 (51.6-83.7) | 72.3 (57.2-82.9) | 0.794 |
| Truncus arteriosus | 69.3 (59.9-76.9) | 78.1 (68.9-84.9) | 75.3 (64.4-83.3) | 70.4 (59.1-79.1) | 0.444 |
| Truncus with IAA | 46.2 (19.2-69.6) | -- ^b^ | -- ^b^ | -- ^b^ | -- ^b^ |
| Truncus without IAA | 72.3 (62.4-79.9) | 77.9 (68.1-85.0) | 75.7 (64.2-83.9) | 71.6 (59.9-80.5) | 0758 |
| Interrupted aortic arch, type B | 61.4 (47.5-72.6) | 80.0 (66.8-88.4) | 76.9 (60.3-87.3) | 83.3 (71.9-90.4) | **0.023** |
| Right arch | -- ^b^ | -- ^b^ | -- ^b^ | -- ^b^ | -- ^b^ |
| Double aortic arch | -- ^b^ | -- ^b^ | -- ^b^ | -- ^b^ | -- ^b^ |
| DORV | 67.0 (61.1-72.3) | 71.4 (66.0-76.1) | 73.1 (67.5-77.9) | 77.2 (71.6-81.9) | 0.063 |
| DORV, TGA (Taussig-Bing) | -- ^b^ | 85.7 (72.4-92.9) | 82.1 (66.0-91.0) | -- ^b^ | -- ^b^ |
| DORV, normal GA/TOF-type | 68.3 (59.3-75.7) | 72.7 (64.0-79.5) | 80.0 (71.0-86.5) | 80.8 (70.1-87.9) | 0.141 |
| DORV, complex | 66.2 (53.6-76.1) | 69.0 (56.9-78.4) | 66.7 (55.0-75.9) | 75.2 (66.2-82.2) | 0.434 |
| DORV, mitral atresia | 50.0 (35.6-62.8) | 60.0 (46.5-71.1) | 63.2 (49.3-74.2) | 71.7 (57.5-81.9) | 0.133 |
| All endocardial defects | 77.5 (74.4-80.3) | 81.2 (78.3-83.7) | 79.6 (76.2-82.5) | 79.3 (76.0-82.1) | 0.314 |
| Primum atrial septal defect | 82.5 (66.8-91.2) | 81.8 (63.9-91.4) | -- ^b^ | 85.0 (69.6-93.0) | -- ^b^ |
| AV septal defect, simple | 83.2 (79.1-86.6) | 88.7 (84.8-91.6) | 88.6 (84.1-91.9) | 88.2 (84.1-91.4) | 0.080 |
| AV septal defect, complex | 69.3 (64.1-73.9) | 74.8 (70.4-78.7) | 71.1 (65.8-75.7) | 70.5 (65.4-75.0) | 0.270 |
| All laterality defects | 83.2 (79.9-85.9) | 83.3 (80.0-86.2) | 87.7 (84.2-90.5) | 85.6 (81.9-88.6) | 0.170 |
| Congenitally corrected TGA | 81.1 (67.8-89.4) | -- ^b^ | -- ^b^ | 85.7 (69.0-93.8) | -- ^b^ |
| dTGA | 89.7 (86.0-92.4) | 89.1 (85.5-91.9) | 94.5 (91.0-96.6) | 93.3 (89.6-95.7) | **0.046** |
| dTGA with VSD | 87.7 (80.9-92.2) | 88.5 (82.5-92.5) | 95.5 (89.5-98.1) | 94.5 (87.3-97.7) | 0.075 |
| dTGA without VSD | 91.5 (86.5-94.7) | 90.9 (85.5-94.3) | 93.2 (87.3-96.4) | 91.2 (85.1-94.9) | 0.885 |
| DILV | 72.3 (65.3-78.0) | 69.1 (61.4-75.6) | 71.4 (62.4-78.7) | 68.8 (59.9-76.1) | 0.846 |
| DILV, L-looping | -- ^b^ | 78.6 (58.4-89.8) | -- ^b^ | -- ^b^ | -- ^b^ |
| **Defect (continued)** | **1999-2004**  **(continued)** | **2005-2009**  **(continued)** | **2010-2013**  **(continued)** | **2014-2017**  **(continued)** | **p-value^a^**  **(continued)** |
| DILV, other | 70.1 (62.5-76.4) | 67.2 (58.5-74.4) | 66.3 (55.9-74.8) | 65.4 (55.4-73.7) | 0.806 |
| Other defect types | -- | -- | -- | -- | -- |
| Aortopulmonary window | -- ^b^ | -- ^b^ | -- ^b^ | -- ^b^ | -- ^b^ |
| Partial anomalous PV return | 75.0 (56.2-86.6) | 84.4 (70.1-92.3) | -- ^b^ | -- ^b^ | -- ^b^ |
| Total anomalous PV return | 86.8 (80.7-91.1) | 85.0 (79.1-89.4) | 87.1 (79.5-92.0) | 87.1 (79.5-92.0 | 0.955 |
| VSD | 96.3 (95.8-96.7) | 96.1 (95.7-96.4) | 97.2 (96.8-97.5) | 97.3 (97.0-97.6) | **<0.0001** |
| VSD, muscular | -- ^b^ | -- ^b^ | 96.9 (95.5-97.9) | 98.3 (98.0-98.6) | -- ^b^ |
| VSD, membranous | -- ^b^ | -- ^b^ | 95.9 (92.7-97.7) | 98.3 (98.0-98.6) | -- ^b^ |
| VSD, other | 96.3 (95.8-96.7) | 96.1 (95.7-96.5) | 97.3 (96.9-97.6) | 94.1 (92.2-95.5) | **<0.0001** |

AV: Atrioventricular; CHD: Congenital heart defects; CI: Confidence interval; D: Day; dTGA: Dextro-transposition of the great arteries; DILV: Double inlet left ventricle; DORV: Double outlet left ventricle; GA: Great arteries; IAA: Interrupted aortic arch; IQR: Interquartile range; IVS: Intact ventricular septum; PV: Pulmonary venous; Synd.: Syndrome; TGA: Transposition of the great arteries; TOF: Tetralogy of Fallot; Truncus: Truncus arteriosus; VSD: Ventricular septal defect.

^a^Bolded p-values correspond to a log-rank p<0.05.

^b^Survival estimates for CHDs with <5 deaths within a given category were not computed or included on statistical analyses.

**SUPPLEMENTAL TABLE 3. Survival to Age 1 Year by Maternal Race/Ethnicity Among Infants with CHD, Texas, 1999-2017 Births^a^**

| **Defect** | **Hispanic** | **Non-Hispanic White** | **Non-Hispanic Black** | **Additional Groups** | **p-value^a^** |
| --- | --- | --- | --- | --- | --- |
| All CHDs | 92.2 (91.9-92.5) | 92.9 (92.5-93.2) | 89.7 (88.9-90.4) | 92.8 (91.7-93.7) | **<0.001** |
| All left-sided lesions | 84.2 (83.0-85.3) | 85.7 (84.5-86.9) | 78.6 (75.6-81.3) | 84.0 (79.3-87.7) | **<0.0001** |
| Arch obstruction, simple | 93.4 (92.0-94.6) | 93.7 (91.9-95.0) | 94.0 (90.1-96.4) | 87.1 (77.9-92.6) | 0.107 |
| Arch obstruction, complex | 84.6 (82.4-86.4) | 86.1 (83.7-88.2) | 78.5 (72.3-83.6) | 84.9 (75.9-90.8) | 0.065 |
| Congenital aortic stenosis | 94.1 (88.3-93.1) | 94.4 (91.2-96.5) | -- ^b^ | -- ^b^ | -- ^b^ |
| Mitral stenosis, simple | 90.8 (86.3-93.9) | 91.1 (85.7-94.6) | 93.5 (85.1-97.2) | -- ^b^ | -- ^b^ |
| Mitral stenosis, complex | 78.1 (82.0-83.1) | 79.6 (71.8-85.4) | 63.6 (49.5-74.8) | -- ^b^ | -- ^b^ |
| Cor triatriatum | -- ^b^ | -- ^b^ | -- ^b^ | -- ^b^ | -- ^b^ |
| Supravalvar aortic stenosis | -- ^b^ | -- ^b^ | -- ^b^ | -- ^b^ | -- ^b^ |
| Hypoplastic left heart synd. | 55.0 (50.6-59.2) | 64.2 (59.7-68.3) | 45.3 (37.1-53.1) | 50.0 (31.9-65.7) | **0.0002** |
| All right-sided lesions | 93.2 (92.2-94.1) | 94.5 (93.4-95.4) | 92.0 (89.7-93.7) | 93.5 (89.7-96.0) | 0.120 |
| Ebstein anomaly | 76.3 (70.2-81.3) | 87.2 (80.5-91.8) | 62.1 (42.1-76.9) | -- ^b^ | -- ^b^ |
| Pulmonary stenosis | 98.3 (97.5-98.8) | 98.5 (97.7-99.1) | 97.4 (95.6-98.5) | -- ^b^ | -- ^b^ |
| Pulmonary atresia, IVS | 75.6 (68.4-81.4) | 77.6 (69.2-84.0) | 72.2 (54.5-84.0) | 77.8 (57.1-89.3) | 0.962 |
| Tricuspid atresia | 82.2 (72.6-88.7) | 84.8 (77.5-89.9) | 68.6 (54.0-79.5) | 80.0 (58.4-91.1) | 0.144 |
| Tricuspid atresia, normal GA | 80.3 (72.9-85.8) | 84.3 (73.1-88.6) | 69.6 (54.1-80.7) | -- ^b^ | -- ^b^ |
| Tricuspid atresia, malposed GA | 78.8 (65.1-87.7) | -- ^b^ | -- ^b^ | -- ^b^ | -- ^b^ |
| All conotruncal defects | 81.6 (79.7-83.4) | 89.0 (87.2-90.6) | 87.7 (84.4-90.3) | 88.5 (83.2-92.2) | **<0.0001** |
| Tetralogy of Fallot | 87.4 (85.0-89.4) | 92.7 (90.6-94.3) | 91.0 (86.9-93.8)) | 90.2 (83.6-94.2) | **0.004** |
| Pulmonary atresia with VSD/TOF | 73.3 (68.0-77.9) | 77.8 (71.0-83.3) | 79.4 (67.1-87.5) | 71.4 (47.2-86.0) | 0.622 |
| Pulmonary atresia, complex | 69.2 (57.7-78.2) | 80.5 (64.8-89.7) | 58.3 (27.0-80.1) | 16.7 (0.8-51.7) | **0.010** |
| Truncus arteriosus | 68.3 (61.4-74.2) | 78.2 (69.6-84.6) | 75.6 (60.2-85.6) | -- ^b^ | -- ^b^ |
| Truncus with IAA | 50.0 (25.9-70.1) | 64.3 (34.3-83.3) | -- ^b^ | -- ^b^ | -- ^b^ |
| Truncus without IAA | 70.1 (62.9-76.1) | 80.0 (71.0-86.5) | 73.2 (56.8-84.1) | -- ^b^ | -- ^b^ |
| Interrupted aortic arch, type B | 69.4 (59.2-77.5) | 81.7 (71.5-88.5) | 83.3 (64.5-92.7) | -- ^b^ | -- ^b^ |
| Right arch | 98.1 (92.6-99.5) | -- ^b^ | -- ^b^ | -- ^b^ | -- ^b^ |
| Double aortic arch | -- ^b^ | -- ^b^ | -- ^b^ | -- ^b^ | -- ^b^ |
| DORV | 69.8 (66.0-73.4) | 76.3 (71.6-80.4) | 69.1 (60.1-76.5) | 74.5 (59.4-84.6) | 0.182 |
| DORV, TGA (Taussig-Bing) | 82.7 (72.6-89.4) | -- ^b^ | -- ^b^ | -- ^b^ | -- ^b^ |
| DORV, normal GA/TOF-type | 73.1 (66.5-78.6) | 78.6 (71.0-84.4) | 72.4 (59.0-82.1) | 68.2 (44.6-83.4) | 0.141 |
| DORV, complex | 69.0 (61.5-75.4) | 69.4 (59.8-77.2) | 69.7 (51.0-82.4) | -- ^b^ | -- ^b^ |
| DORV, mitral atresia | 57.5 (48.4-65.5) | 70.6 (58.2-79.9) | 52.6 (28.7-71.9) | -- ^b^ | -- ^b^ |
| All endocardial defects | 75.4 (73.0-77.7) | 83.7 (81.4-85.7) | 80.7 (76.2-84.3) | 77.4 (68.2-84.2) | 0.314 |
| Primum atrial septal defect | 85.7 (74.3-92.3) | 85.2 (73.6-92.0) | -- ^b^ | -- ^b^ | -- ^b^ |
| AV septal defect, simple | 83.4 (79.9-86.3) | 90.0 (87.1-92.2) | 89.0 (83.3-92.8) | 84.3 (71.1-91.8) | **0.012** |
| AV septal defect, complex | 67.9 (64.3-71.3) | 76.8 (73.0-80.2) | 70.5 (62.9-76.8) | 72.3 (57.2-82.9) | 0.270 |
| All laterality defects | 83.1 (80.6-85.3) | 87.8 (85.3-89.9) | 78.0 (70.9-83.6) | 87.1 (77.9-92.6) | 0.170 |
| Congenitally corrected TGA | 87.5 (77.4-93.3) | 88.2 (77.8-93.9) | -- ^b^ | -- ^b^ | -- ^b^ |
| dTGA | 91.1 (88.5-93.2) | 92.5 (89.9-94.4) | 87.9 (79.6-92.9) | 88.7 (77.8-94.5) | **0.046** |
| dTGA with VSD | 91.2 (86.8-94.2) | 92.8 (88.1-95.7) | 85.1 (71.3-92.6) | -- ^b^ | -- ^b^ |
| dTGA without VSD | 91.3 (87.1-94.1) | 92.1 (88.3-94.7) | -- ^b^ | -- ^b^ | -- ^b^ |
| DILV | 68.4 (63.2-73.1) | 75.5 (68.7-81.1) | 63.0 (48.7-74.3) | -- ^b^ | -- ^b^ |
| DILV, L-looping | 81.4 (66.2-90.2) | 89.1 (75.8-95.3) | -- ^b^ | -- ^b^ | -- ^b^ |
| **Defect (continued)** | **Hispanic**  **(continued)** | **Non-Hispanic White**  **(continued)** | **Non-Hispanic Black**  **(continued)** | **Non-Hispanic Additional**  **(continued)** | **p-value^a^**  **(continued)** |
| DILV, other | 66.6 (60.9-71.6) | 71.1 (62.9-77.8) | 61.2 (46.2-73.2) | -- ^b^ | -- ^b^ |
| Other defect types | -- | -- | -- | -- | -- |
| Aortopulmonary window | -- ^b^ | -- ^b^ | -- ^b^ | -- ^b^ | -- ^b^ |
| Partial anomalous PV return | 86.7 (76.6-92.6) | 91.2 (80.2-96.3) | 80.8 (59.8-91.5) | -- ^b^ | -- ^b^ |
| Total anomalous PV return | 87.3 (83.6-90.3) | 85.5 (79.2-90.1) | 82.1 (66.0-91.0) | 82.9 (65.8-91.9) | 0.955 |
| VSD | 96.9 (96.6-97.1) | 96.9 (96.6-97.2) | 95.0 (94.1-95.7) | 97.3 (96.4-98.0) | **<0.0001** |
| VSD, muscular | 98.4 (98.0-98.7) | -- ^b^ | -- ^b^ | -- ^b^ | 0.181 |
| VSD, membranous | -- ^b^ | 95.9 (93.8-97.3) | 91.5 (87.3-94.4) | -- ^b^ | -- ^b^ |
| VSD, other | 96.6 (96.3-96.8) | 96.6 (96.2-97.0) | 94.8 (93.8-95.6) | 96.8 (95.5-97.8) | **<0.0001** |

AV: Atrioventricular; CHD: Congenital heart defects; CI: Confidence interval; D: Day; dTGA: Dextro-transposition of the great arteries; DILV: Double inlet left ventricle; DORV: Double outlet left ventricle; GA: Great arteries; IAA: Interrupted aortic arch; IQR: Interquartile range; IVS: Intact ventricular septum; PV: Pulmonary venous; Synd.: Syndrome; TGA: Transposition of the great arteries; TOF: Tetralogy of Fallot; Truncus: Truncus arteriosus; VSD: Ventricular septal defect.

^a^Bolded p-values correspond to a log-rank p<0.05.

^b^Survival estimates for CHDs with <5 deaths within a given category were not computed or included on statistical analyses.

**SUPPLEMENTAL TABLE 4. Survival to Age 1 Year by Infant Sex Among Infants with CHD, Texas, 1999-2017 Births^a^**

| **Defect** | **Male** | **Female** | **p-value^a^** |
| --- | --- | --- | --- |
| All CHDs | 91.9 (91.5-92.2) | 92.6 (92.3-92.8) | **0.001** |
| All left-sided lesions | 85.3 (84.3-86.3) | 82.7 (81.4-83.9) | **0.001** |
| Arch obstruction, simple | 93.2 (91.8-94.3) | 93.7 (92.0-95.0) | 0.614 |
| Arch obstruction, complex | 86.2 (84.3-88.0) | 82.7 (80.3-84.8) | **0.016** |
| Congenital aortic stenosis | 94.7 (92.3-96.4) | 90.1 (85.5-93.3) | **0.021** |
| Mitral stenosis, simple | 91.1 (86.9-94.0) | 92.2 (88.1-95.0) | 0.658 |
| Mitral stenosis, complex | 79.1 (73.0-84.0) | 74.3 (67.9-79.6) | 0.261 |
| Cor triatriatum | -- ^b^ | -- ^b^ | -- ^b^ |
| Supravalvar aortic stenosis | -- ^b^ | -- ^b^ | -- ^b^ |
| Hypoplastic left heart synd. | 61.3 (57.7-64.7) | 50.7 (45.9-55.3) | **0.0002** |
| All right-sided lesions | 92.9 (91.8-93.8) | 94.0 (93.1-94.7) | 0.107 |
| Ebstein anomaly | 81.0 (74.9-85.7) | 78.6 (72.5-83.5) | 0.489 |
| Pulmonary stenosis | 97.6 (96.7-98.3) | 98.7 (98.1-99.1) | **0.013** |
| Pulmonary atresia, IVS | 80.9 (74.3-86.0) | 71.8 (64.5-77.8) | **0.038** |
| Tricuspid atresia | 81.4 (75.7-86.0) | 78.6 (72.0-83.8) | 0.457 |
| Tricuspid atresia, normal GA | 81.8 (74.8-86.9) | 76.7 (69.0-82.6) | 0.242 |
| Tricuspid atresia, malposed GA | 80.6 (68.4-88.5) | 86.1 (69.8-94.0) | 0.461 |
| All conotruncal defects | 86.4 (84.9-87.9) | 84.2 (82.4-85.8) | 0.055 |
| Tetralogy of Fallot | 90.5 (88.6-92.0) | 89.5 (87.3-91.3) | 0.449 |
| Pulmonary atresia with VSD/TOF | 75.6 (70.5-80.1) | 74.9 (69.2-79.7) | 0.895 |
| Pulmonary atresia, complex | 73.5 (61.3-82.4) | 65.7 (53.4-75.5) | 0.312 |
| Truncus arteriosus | 75.5 (68.7-81.1) | 71.0 (64.0-76.8) | 0.338 |
| Truncus with IAA | -- ^b^ | 56.5 (34.3-73.8) | -- ^b^ |
| Truncus without IAA | 75.9 (68.8-81.5) | 72.9 (65.6-79.0) | 0.577 |
| Interrupted aortic arch, type B | 75.8 (65.6-83.4) | 75.4 (66.9-82.0) | 0.900 |
| Right arch | -- ^b^ | 91.1 (82.3-95.7) | -- ^b^ |
| Double aortic arch | 96.0 (91.3-98.2) | -- ^b^ | -- ^b^ |
| DORV | 75.6 (72.1-78.7) | 67.0 (62.5-71.1) | **0.0016** |
| DORV, TGA (Taussig-Bing) | 89.9 (82.0-94.4) | 77.8 (60.4-88.2) | **0.044** |
| DORV, normal GA/TOF-type | 76.9 (71.0-81.7) | 71.8 (64.9-77.6) | 0.292 |
| DORV, complex | 74.2 (67.5-79.8) | 63.4 (54.7-71.0) | **0.040** |
| DORV, mitral atresia | 63.6 (54.4-71.5) | 58.6 (48.2-67.6) | 0.470 |
| All endocardial defects | 79.0 (76.8-81.1) | 79.8 (77.7-81.7) | 0.634 |
| Primum atrial septal defect | 87.8 (77.9-93.5) | 82.5 (72.2-89.2) | 0.345 |
| AV septal defect, simple | 86.5 (83.4-89.1) | 87.2 (84.6-89.5) | 0.756 |
| AV septal defect, complex | 71.8 (68.3-75.0) | 71.6 (68.2-74.8) | 0.970 |
| All laterality defects | 87.5 (85.6-89.2) | 79.3 (76.0-82.1) | **<0.0001** |
| Congenitally corrected TGA | 91.3 (83.9-95.4) | 75.9 (62.2-85.2) | **0.006** |
| dTGA | 92.3 (90.3-93.9) | 89.3 (85.8-91.9) | 0.073 |
| dTGA with VSD | 91.5 (87.9-94.1) | 89.5 (83.9-93.3) | 0.452 |
| dTGA without VSD | 93.1 (90.3-95.1) | 88.0 (82.4-92.0) | **0.035** |
| DILV | 75.3 (70.5-79.4) | 63.1 (56.6-68.9) | **0.002** |
| DILV, L-looping | 88.3 (77.1-94.3) | 80.6 (63.5-90.2) | 0.294 |
| **Defect (continued)** | **Male** | **Female** | **p-value^a^** |
| DILV, other | 72.7 (67.2-77.3) | 60.0 (52.9-66.4) | **0.005** |
| Other defect types | -- | -- | -- |
| Aortopulmonary window | -- ^b^ | -- ^b^ | -- ^b^ |
| Partial anomalous PV return | 86.6 (78.0-92.0) | 89.7 (79.6-95.0) | 0.571 |
| Total anomalous PV return | 86.0 (82.4-89.0) | 86.8 (81.2-90.8) | 0.781 |
| VSD | 96.5 (96.2-96.8) | 96.9 (96.6-97.1) | **0.046** |
| VSD, muscular | 97.9 (97.3-98.3) | 98.5 (98.0-98.8) | 0.050 |
| VSD, membranous | 95.4 (93.8-96.5) | 94.6 (93.0-95.9) | 0.458 |
| VSD, other | 96.3 (95.9-96.6) | 96.6 (96.3-96.9) | 0.139 |

AV: Atrioventricular; CHD: Congenital heart defects; CI: Confidence interval; D: Day; dTGA: Dextro-transposition of the great arteries; DILV: Double inlet left ventricle; DORV: Double outlet left ventricle; GA: Great arteries; IAA: Interrupted aortic arch; IQR: Interquartile range; IVS: Intact ventricular septum; PV: Pulmonary venous; Synd.: Syndrome; TGA: Transposition of the great arteries; TOF: Tetralogy of Fallot; Truncus: Truncus arteriosus; VSD: Ventricular septal defect.

^a^Bolded p-values correspond to a log-rank p<0.05.

^b^Survival estimates for CHDs with <5 deaths within a given category were not computed or included on statistical analyses.

**SUPPLEMENTAL TABLE 5. Survival to Age 1 Year by Gestational Age Among Infants with CHD, Texas, 1999-2017 Births^a,b^**

| **Defect** | **<32 weeks** | **32 to <37 weeks** | **>37 weeks** | **p-value^a^** |
| --- | --- | --- | --- | --- |
| All CHDs | 80.8 (79.7-82.0) | 88.2 (87.6-88.8) | 94.3 (94.0-94.5) | **<0.0001** |
| All left-sided lesions | 67.8 (63.3-71.9) | 78.5 (76.4-80.5) | 86.8 (86.0-87.6) | **<0.0001** |
| Arch obstruction, simple | 78.6 (71.4-84.2) | 91.0 (88.3-93.1) | 95.3 (94.3-96.2) | **<0.0001** |
| Arch obstruction, complex | 58.9 (49.7-66.9) | 75.2 (71.1-78.7) | 88.9 (87.4-90.3) | **<0.0001** |
| Congenital aortic stenosis | 76.6 (61.7-86.3) | 88.4 (81.2-93.0) | 95.5 (93.4-96.9) | **<0.0001** |
| Mitral stenosis, simple | 81.8 (66.9-90.5) | 87.8 (79.4-92.9) | 93.9 (90.9-95.9) | **0.006** |
| Mitral stenosis, complex | 56.5 (34.3-73.8) | 67.1 (55.8-76.1) | 80.6 (75.9-84.6) | **0.0008** |
| Cor triatriatum | -- ^c^ | -- ^c^ | -- ^c^ | -- ^c^ |
| Supravalvar aortic stenosis | -- ^b^ | -- ^c^ | -- ^c^ | -- ^c^ |
| Hypoplastic left heart synd. | -- ^c^ | 39.1 (31.6-46.6) | 61.5 (58.4-64.5) | -- ^c^ |
| All right-sided lesions | 88.3 (85.8-90.4) | 90.1 (88.1-91.8) | 95.2 (94.5-95.9) | **<0.0001** |
| Ebstein anomaly | 25.0 (9.1-44.9) | 64.6 (53.0-74.0) | 86.7 (82.5-89.9) | **<0.0001** |
| Pulmonary stenosis | 93.9 (91.7-95.6) | 98.1 (96.7-98.9) | 99.2 (98.8-99.5) | **<0.0001** |
| Pulmonary atresia, IVS | 40.0 (21.3-58.1) | 69.2 (56.5-78.9) | 81.0 (75.7-85.2) | **<0.0001** |
| Tricuspid atresia | -- ^c^ | 63.2 (50.6-73.4) | 87.2 (83.1-90.4) | -- ^c^ |
| Tricuspid atresia, normal GA | -- ^c^ | 62.5 (48.5-73.7) | 86.2 (81.1-90.0) | -- ^c^ |
| Tricuspid atresia, malposed GA | -- ^c^ | -- ^c^ | 90.2 (81.4-95.0) | -- ^c^ |
| All conotruncal defects | 56.4 (49.3-62.9) | 79.7 (76.7-82.4) | 89.0 (87.8-90.1) | **<0.0001** |
| Tetralogy of Fallot | 62.0 (52.2- 70.4) | 84.5 (80.5-87.7) | 93.2 (91.9-94.4) | **<0.0001** |
| Pulmonary atresia with VSD/TOF | 32.3 (16.9-48.6) | 66.7 (57.8-74.1) | 81.5 (77.4-84.9) | **<0.0001** |
| Pulmonary atresia, complex | -- ^c^ | 53.6 (33.8-69.8) | 78.4 (69.1-85.2) | -- ^c^ |
| Truncus arteriosus | 30.0 (12.3-50.1) | 76.7 (65.2-84.8) | 75.3 (69.9-79.9) | **<0.0001** |
| Truncus with IAA | -- ^c^ | -- ^c^ | 59.4 (40.5-74.0) | -- ^c^ |
| Truncus without IAA | 31.6 (12.9-52.2) | 75.4 (63.4-83.9) | 77.3 (71.7-82.0) | **<0.0001** |
| Interrupted aortic arch, type B | -- ^c^ | 54.8 (38.7-68.3) | 81.7 (75.0-86.7) | -- ^c^ |
| Right arch | -- ^c^ | -- ^c^ | -- ^c^ | -- ^c^ |
| Double aortic arch | -- ^c^ | -- ^c^ | -- ^c^ | -- ^c^ |
| DORV | 38.2 (26.8-49.6) | 61.9 (54.6-68.3) | 77.1 (74.2-79.8) | **<0.0001** |
| DORV, TGA (Taussig-Bing) | -- ^c^ | -- ^c^ | 87.3 (79.8-92.1) | -- ^c^ |
| DORV, normal GA/TOF-type | 50.0 (32.4-65.3) | 67.5 (56.1-76.6) | 79.1 (74.2-83.1) | **<0.0001** |
| DORV, complex | 20.8 (7.6-38.5) | 58.0 (43.2-70.2) | 77.0 (71.3-81.6) | **<0.0001** |
| DORV, mitral atresia | -- ^c^ | 50.0 (35.3-63.1) | 66.3 (58.4-72.9) | -- ^c^ |
| All endocardial defects | 43.5 (35.4-51.4) | 70.8 (67.1-74.2) | 84.4 (82.8-85.9) | **<0.0001** |
| Primum atrial septal defect | 47.1 (23.0-68.0) | 82.1 (66.0-91.0) | 92.9 (85.6-96.5) | **<0.0001** |
| AV septal defect, simple | 47.5 (34.4-59.5) | 78.7 (73.5-83.0) | 91.9 (89.9-93.4) | **<0.0001** |
| AV septal defect, complex | 38.2 (26.8-49.6) | 61.5 (55.8-66.8) | 76.6 (74.0-79.1) | **<0.0001** |
| All laterality defects | 42.2 (27.8-56.0) | 71.2 (65.2-76.3) | 87.9 (86.2-89.3) | **<0.0001** |
| Congenitally corrected TGA | -- ^c^ | 76.9 (55.7-88.9) | 88.4 (81.5-92.8) | -- ^c^ |
| dTGA | 50.0 (29.9-67.2) | 81.7 (74.3-87.1) | 93.6 (91.9-94.9) | **<0.0001** |
| dTGA with VSD | -- ^c^ | 84.1 (73.1-90.8) | 93.0 (90.1-95.0) | -- ^c^ |
| dTGA without VSD | 57.1 (28.4-78.0) | 79.0 (66.6-87.2) | 93.9 (91.5-95.6) | **<0.0001** |
| DILV | 29.4 (10.7-51.1) | 53.3 (42.6-62.8) | 75.2 (71.1-78.8) | **<0.0001** |
| **Defect (continued)** | **<32 weeks (continued)** | **32 to <37 weeks**  **(continued)** | **>37 weeks**  **(continued)** | **p-value^a^**  **(continued)** |
| DILV, L-looping | -- ^c^ | -- ^c^ | 86.9 (77.6-92.5) | -- ^c^ |
| DILV, other | 29.4 (10.7-51.1) | 49.4 (38.0-59.8) | 72.8 (68.2-76.8) | **<0.0001** |
| Other defect types | -- | -- | -- | -- |
| Aortopulmonary window | -- ^c^ | -- ^c^ | -- ^c^ | -- ^c^ |
| Partial anomalous PV return | 70.6 (43.1-86.6) | -- ^c^ | 90.6 (83.7-94.7) | -- ^c^ |
| Total anomalous PV return | -- ^c^ | 73.4 (62.2-81.8) | 89.3 (86.4-91.7) | -- ^c^ |
| VSD | 86.5 (85.2-87.7) | 94.9 (94.3-95.4) | 98.2 (98.0-98.3) | **<0.0001** |
| VSD, inlet | -- ^c^ | -- ^c^ | -- ^c^ | -- ^c^ |
| VSD, muscular | 90.2 (87.5-92.3) | 97.3 (96.2-98.0) | 99.1 (98.8-99.3) | **<0.0001** |
| VSD, membranous  VSD, other | 81.6 (74.4-86.9)  85.8 (84.3-87.2) | 94.4 (91.5-96.3)  94.3 (93.6-95.0) | 96.6 (95.5-97.4)  98.0 (97.8-98.2) | **<0.0001**  **<0.0001** |

AV: Atrioventricular; CHD: Congenital heart defects; CI: Confidence interval; D: Day; dTGA: Dextro-transposition of the great arteries; DILV: Double inlet left ventricle; DORV: Double outlet left ventricle; GA: Great arteries; IAA: Interrupted aortic arch; IQR: Interquartile range; IVS: Intact ventricular septum; PV: Pulmonary venous; Synd.: Syndrome; TGA: Transposition of the great arteries; TOF: Tetralogy of Fallot; Truncus: Truncus arteriosus; VSD: Ventricular septal defect.

^a^Infants were evaluated by categorized gestational age at birth.

^b^Bolded p-values correspond to a log-rank p<0.05.

^c^Survival estimates for CHDs with <5 deaths within a given category were not computed or included on statistical analyses.

**SUPPLEMENTAL TABLE 6. Survival to Age 1 Year by Low Birthweight Among Infants with CHD, Texas, 1999-2017 Births^a^**

| **Defect** | **Low Birthweight** | **Not Low Birthweight** | | **p-value^a^** | |
| --- | --- | --- | --- | --- | --- |
| All CHDs | 82.4 (81.7-83.0) | 95.3 (95.1-95.5) | **<0.0001** | | |
| All left-sided lesions | 70.8 (68.8-72.8) | 88.4 (87.6-89.1) | **<0.0001** | | |
| Arch obstruction, simple | 83.9 (80.7-86.6) | 96.2 (95.3-96.9) | **<0.0001** | | |
| Arch obstruction, complex | 69.2 (65.4-72.7) | 90.0 (88.5-91.3) | **<0.0001** | | |
| Congenital aortic stenosis | 79.0 (71.4-84.8) | 96.7 (94.9-97.9) | **<0.0001** | | |
| Mitral stenosis, simple | 84.5 (77.0-89.7) | 94.1 (91.2-96.1) | **0.001** | | |
| Mitral stenosis, complex | 54.4 (45.3-62.6) | 86.0 (81.5-89.4) | **<0.0001** | | |
| Cor triatriatum | -- ^b^ | -- ^b^ | -- ^b^ | | |
| Supravalvar aortic stenosis | 78.3 (55.4-90.3) | -- ^b^ | -- ^b^ | | |
| Hypoplastic left heart synd. | 33.8 (27.5-40.2) | 62.7 (59.5-65.7) | **<0.0001** | | |
| All right-sided lesions | 86.5 (84.7-88.1) | 96.3 (95.6-96.8) | **<0.0001** | | |
| Ebstein anomaly | 53.9 (43.1-63.6) | 86.7 (82.6-89.9) | **<0.0001** | | |
| Pulmonary stenosis | 95.9 (94.5-96.9) | 99.3 (98.9-99.5) | **<0.0001** | | |
| Pulmonary atresia, IVS | 50.5 (40.0-60.2) | 85.2 (80.3-89.0) | **<0.0001** | | |
| Tricuspid atresia | 56.3 (46.6-64.8) | 89.1 (84.2-92.6) | **<0.0001** | | |
| Tricuspid atresia, normal GA | 55.2 (44.2-64.7) | 89.7 (80.5-94.7) | **<0.0001** | | |
| Tricuspid atresia, malposed GA | 59.1 (36.1-76.2) | 89.5 (80.1-94.6) | **0.0005** | | |
| All conotruncal defects | 73.7 (71.0-76.2) | 90.4 (89.2-91.5) | **<0.0001** | | |
| Tetralogy of Fallot | 79.1 (75.7-82.2) | 94.5 (93.2-95.6) | **<0.0001** | | |
| Pulmonary atresia with VSD/TOF | 58.8 (51.3-65.5) | 83.4 (79.2-86.7) | **<0.0001** | | |
| Pulmonary atresia, complex | 40.0 (25.0-54.6) | 81.6 (72.4-88.0) | **<0.0001** | | |
| Truncus arteriosus | 68.6 (58.8-76.5) | 75.3 (69.7-79.9) | 0.099 | | |
| Truncus with IAA | -- ^b^ | 62.1 (42.1-76.9) | -- ^b^ | | |
| Truncus without IAA | 69.1 (58.8-77.2) | 76.8 (71.1-81.6) | 0.074 | | |
| Interrupted aortic arch, type B | 54.2 (40.8-65.9) | 84.1 (77.4-88.9) | **<0.0001** | | |
| Right arch | 86.8 (74.3-93.5) | -- ^b^ | -- ^b^ | | |
| Double aortic arch | 91.4 (81.9-96.1) | -- ^b^ | -- ^b^ | | |
| DORV | 46.8 (41.3-52.1) | 82.9 (80.1-85.4) | **<0.0001** | | |
| DORV, TGA (Taussig-Bing) | 66.7 (40.4-83.4) | 89.7 (82.6-94.0) | **0.002** | | |
| DORV, normal GA/TOF-type | 51.7 (43.3-59.4) | 87.3 (82.8-90.7) | **<0.0001** | | |
| DORV, complex | 48.3 (37.6-58.2) | 77.4 (69.7-83.4) | **<0.0001** | | |
| DORV, mitral atresia | 30.1 (20.1-40.8) | 77.4 (69.7-83.4) | **<0.0001** | | |
| All endocardial defects | 63.9 (60.4-67.2) | 85.0 (83.4-86.4) | **<0.0001** | | |
| Primum atrial septal defect | 68.8 (53.6-79.8) | 92.4 (85.3-96.1) | **0.0001** | | |
| AV septal defect, simple | 70.6 (65.3-75.2) | 92.5 (90.7-94.0) | **<0.0001** | | |
| AV septal defect, complex | 56.9 (51.7-61.7) | 77.1 (74.4-79.5) | **<0.0001** | | |
| All laterality defects | 62.4 (56.5-67.8) | 88.4 (86.8-89.8) | **<0.0001** | | |
| Congenitally corrected TGA | 66.7 (42.5-82.5) | 89.0 (82.4-93.2) | **0.004** | | |
| dTGA | 69.7 (61.8-76.4) | 94.4 (92.8-95.6) | **<0.0001** | | |
| dTGA with VSD | 69.6 (57.2-79.0) | 94.4 (91.8-96.2) | **<0.0001** | | |
| dTGA without VSD | 68.2 (55.5-78.0) | 94.3 (92.1-96.0) | **<0.0001** | | |
| DILV | 51.4 (41.6-60.3) | 74.9 (70.9-78.6) | **<0.0001** | | |
| DILV, L-looping | -- ^b^ | 88.5 (79.7-93.6) | -- ^b^ | | |
| **Defect (continued)** | **Low Birthweight**  **(continued)** | **Not Low Birthweight**  **(continued)** | | | **p-value^a^**  **(continued)** |
| DILV, other | 50.0 (39.9-59.3) | 72.0 (67.3-76.1) | **<0.0001** | | |
| Other defect types | -- | -- | -- | | |
| Aortopulmonary window | 72.2 (45.6-87.4) | -- ^b^ | -- ^b^ | | |
| Partial anomalous PV return | 72.0 (57.4-82.4) | 94.8 (88.8-97.6) | **<0.0001** | | |
| Total anomalous PV return | 67.4 (56.8-75.9) | 89.5 (86.6-91.8) | **<0.0001** | | |
| VSD | 89.8 (89.2-90.5) | 98.8 (98.6-98.9) | **<0.0001** | | |
| VSD, muscular | -- ^b^ | 99.2 (98.9-99.4) | -- ^b^ | | |
| VSD, membranous | 87.5 (84.4-90.0) | 98.2 (97.3-98.8) | **<0.0001** | | |
| VSD, other | 88.9 (88.1-89.7) | 98.7 (98.5-98.8) | **<0.0001** | | |

AV: Atrioventricular; CHD: Congenital heart defects; CI: Confidence interval; D: Day; dTGA: Dextro-transposition of the great arteries; DILV: Double inlet left ventricle; DORV: Double outlet left ventricle; GA: Great arteries; IAA: Interrupted aortic arch; IQR: Interquartile range; IVS: Intact ventricular septum; PV: Pulmonary venous; Synd.: Syndrome; TGA: Transposition of the great arteries; TOF: Tetralogy of Fallot; Truncus: Truncus arteriosus; VSD: Ventricular septal defect.

^a^Bolded p-values correspond to a log-rank p<0.05.

^b^Survival estimates for CHDs with <5 deaths within a given category were not computed or included on statistical analyses.

**SUPPLEMENTAL TABLE 7. Infantile Survival by Genetic and Extracardiac Defect Status Among Infants with CHD, Texas, 1999-2017 Births^a^**

| **Defect** | **Genetic** | **Non-genetic with extracardiac defects** | **Non-genetic without extracardiac defects** | | **p-value^a^** |
| --- | --- | --- | --- | --- | --- |
| All CHDs | 76.7 (75.9-77.5) | 86.1 (85.3-86.8) | 97.0 (96.8-97.1) | | **<0.0001** |
| All left-sided lesions | 70.0 (68.5-72.8) | 79.7 (77.7-81.6) | | 90.5 (89.7-91.3) | **<0.0001** |
| Arch obstruction, simple | 84.3 (80.7-87.3) | 87.7 (84.6-90.2) | | 97.9 (97.1-98.5) | **<0.0001** |
| Arch obstruction, complex | 67.1 (63.3-70.7) | 81.9 (78.4-84.9) | | 94.3 (92.9-95.5) | **<0.0001** |
| Congenital aortic stenosis | 76.1 (64.3-84.4) | 85.2 (75.9-91.1) | | 96.6 (94.8-97.8) | **<0.0001** |
| Mitral stenosis, simple | 85.9 (79-90.7) | 90.5 (82.6-95.0) | | 95.1 (91.7-97.1) | **0.0052** |
| Mitral stenosis, complex | 58.5 (50.9-65.4) | 77.4 (64.9-86.0) | | 93.6 (89.0-96.3) | **<0.0001** |
| Cor triatriatum | -- ^b^ | -- ^b^ | | -- ^b^ | -- ^b^ |
| Supravalvar aortic stenosis | -- ^b^ | -- ^b^ | | -- ^b^ | -- ^b^ |
| Hypoplastic left heart synd. | 34.8 (26.9-42.8) | 56.4 (50.4-62) | | 61.8 (58.3-65.2) | **<0.0001** |
| All right-sided lesions | 81.1 (77.6-84.1) | 87.8 (85.2-90.0) | | 96.0 (95.4-96.5) | **<0.0001** |
| Ebstein anomaly | 73.9 (58.7-84.3) | 72.4 (60.8-81.0) | | 82.6 (77.7-86.4) | 0.119 |
| Pulmonary stenosis | 91.3 (87.1-94.1) | 96.0 (93.4-97.5) | | 99.2 (98.8-99.4) | **<0.0001** |
| Pulmonary atresia, IVS | 51.2 (35.5-64.8) | 69.7 (58.1-78.7) | | 82.7 (77.3-87.0) | **<0.0001** |
| Tricuspid atresia | 70.8 (55.8-81.6) | 78.2 (67.3-85.8) | | 82.3 (77.3-86.3) | 0.192 |
| Tricuspid atresia, normal GA | 67.7 (49.2-80.6) | 78.1 (65.9-86.4) | | 81.5 (75.6-86.1) | 0.191 |
| Tricuspid atresia, malposed GA | -- ^b^ | -- ^b^ | | 84.3 (73.4-91.0) | -- ^b^ |
| All conotruncal defects | 74.6 (72.0-76.9) | 82.7 (79.8-85.2) | | 94.2 (93.0-95.2) | **<0.0001** |
| Tetralogy of Fallot | 77.9 (74.4-81.0) | 87.3 (83.2-90.5) | | 97.1 (96.0-98.0) | **<0.0001** |
| Pulmonary atresia with VSD/TOF | 58.8 (51.6-65.3) | 56.6 (68.1-83.1) | | 88.1 (83.3-91.6) | **<0.0001** |
| Pulmonary atresia, complex | 56.3 (41.2-68.9) | 61.8 (43.4-75.7) | | 85.7 (73.5-92.6) | **0.0031** |
| Truncus arteriosus | 69.9 (62.0-76.5) | 64.4 (53.4-73.4) | | 82.3 (74.9-87.7) | **0.0051** |
| Truncus with IAA | -- ^b^ | 57.1 (28.4-78.0) | | 50.0 (18.4-75.3) | -- ^b^ |
| Truncus without IAA | 69.3 (60.9-76.2) | 65.8 (53.7-75.4) | | 84.7 (77.3-89.9) | **0.0024** |
| Interrupted aortic arch, type B | 75.4 (67.0-81.9) | 61.1 (43.3-74.8) | | 86.3 (73.4-93.2) | **0.027** |
| Right arch | 50.0 (18.4-75.3) | -- ^b^ | | -- ^b^ | -- ^b^ |
| Double aortic arch | -- ^b^ | -- ^b^ | | -- ^b^ | -- ^b^ |
| DORV | 48.4 (43.2-53.4) | 74.2 (68.1-79.3) | | 87.9 (84.8-90.5) | **<0.0001** |
| DORV, TGA (Taussig-Bing) | 72.4 (52.3-85.1) | -- ^b^ | | 92.7 (84.4-96.6) | -- ^b^ |
| DORV, normal GA/TOF-type | 51.2 (43.3-58.6) | 80.5 (70.5-87.4) | | 92.9 (88.0-95.8) | **<0.0001** |
| DORV, complex | 48.4 (38.1-58.0) | 66.2 (53.9-75.9) | | 84.0 (77.3-88.8) | **<0.0001** |
| DORV, mitral atresia | 33.3 (23.2-43.8) | 70.4 (56.3-80.7) | | 80.7 (70.8-87.5) | **<0.0001** |
| All endocardial defects | 79.5 (77.7-81.1) | 63.8 (57.8-69.2) | | 87.3 (84.1-89.8) | **<0.0001** |
| Primum atrial septal defect | 77.5 (65.9-85.5) | -- ^b^ | | -- ^b^ | -- ^b^ |
| AV septal defect, simple | 86.1 (83.9-88.1) | 84.5 (72.3-91.6) | | 92.8 (87.6-95.8) | 0.059 |
| AV septal defect, complex | 71.7 (68.7-74.5) | 56.0 (48.7-62.6) | | 82.4 (77.4-86.4) | **<0.0001** |
| All laterality defects | 59.9 (54.1-65.2) | 79.4 (74.6-83.3) | | 91.4 (89.8-92.7) | **<0.0001** |
| Congenitally corrected TGA | 68.4 (42.8-84.4) | -- ^b^ | | 89.3 (81.9-93.8) | -- ^b^ |
| dTGA | 68.1 (56.0-77.5) | 89.3 (84.0-92.9) | | 93.4 (91.7-94.8) | **<0.0001** |
| dTGA with VSD | 61.9 (45.5-74.6) | 89.6 (80.3-94.7) | | 94.3 (91.4-96.2) | **<0.0001** |
| dTGA without VSD | 75.0 (52.6-87.9) | 89.9 (82.0-94.4) | | 92.7 (90.1-94.7) | **0.0035** |
| DILV | 55.8 (48.8-62.3) | 62.3 (52.7-70.5) | | 84.8 (80.0-88.6) | **<0.0001** |
| **Defect**  **(continued)** | **Genetic**  **(continued)** | **Non-genetic with extracardiac defects**  **(continued)** | | **Non-genetic without extracardiac defects**  **(continued)** | **p-value^a^**  **(continued)** |
| DILV, L-looping | 64.0 (42.2-79.4) | -- ^b^ | | -- ^b^ | -- ^b^ |
| DILV, other | 54.7 (47.2-61.6) | 61.2 (51.1-69.8) | | 81.5 (75.6-86.1) | **<0.0001** |
| Other defect types | -- | -- | | -- | -- |
| Aortopulmonary window | -- ^b^ | -- ^b^ | | -- ^b^ | -- ^b^ |
| Partial anomalous PV return | 51.5 (33.5-66.9) | -- ^b^ | | -- ^b^ | -- ^b^ |
| Total anomalous PV return | 69.2 (54.8-79.9) | 75.8 (67.4-82.3) | | 91.3 (88.2-93.5) | **<0.0001** |
| VSD | 82.5 (81.3-83.7) | 93.1 (92.2-93.8) | | 99.0 (98.9-99.1) | **<0.0001** |
| VSD, muscular | 88.7 (85.5-91.2) | 95.2 (93.4-96.5) | | 99.3 (99.0-99.5) | **<0.0001** |
| VSD, membranous | 84.4 (80.6-87.5) | 90.6 (85.7-93.9) | | 99.1 (98.4-99.5) | **<0.0001** |
| VSD, other | 81.3 (79.8-82.6) | 92.6 (91.5-93.6) | | 99.0 (98.8-99.1) | **<0.0001** |

AV: Atrioventricular; CHD: Congenital heart defects; CI: Confidence interval; D: Day; dTGA: Dextro-transposition of the great arteries; DILV: Double inlet left ventricle; DORV: Double outlet left ventricle; GA: Great arteries; IAA: Interrupted aortic arch; IQR: Interquartile range; IVS: Intact ventricular septum; PV: Pulmonary venous; Synd.: Syndrome; TGA: Transposition of the great arteries; TOF: Tetralogy of Fallot; Truncus: Truncus arteriosus; VSD: Ventricular septal defect.

^a^Bolded p-values correspond to a log-rank p<0.05.

^b^Survival estimates for CHDs with <5 deaths within a given category were not computed or included on statistical analyses.

**SUPPLEMENTAL FIGURE 1. Longitudinal Changes in Survival to Age 1 Year by Birth Year Among Infants with CHD, Texas, 1999-2017^a^**

^
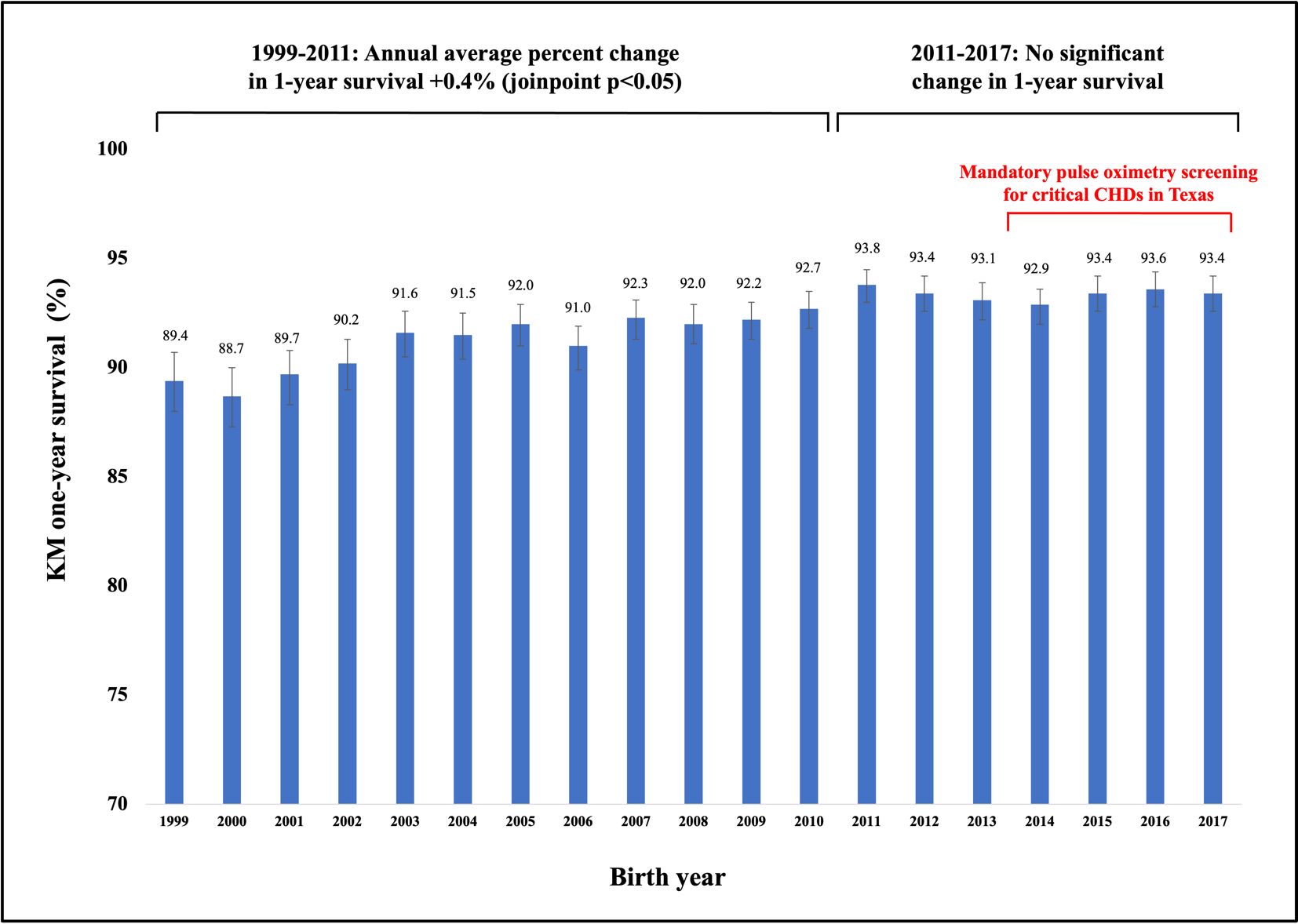
^

^a^CHD: Congenital heart defects; KM: Kaplan-Meier. Error bars correspond to 95% KM confidence limits. Percentage infant mortality (%) by year is provided above each confidence bar.

**SUPPLEMENTAL FIGURE 2. Longitudinal Changes in Survival to Age 1 Year by Birth Year Among Infants with Critical CHD, Texas, 1999-2017 Births^a^**


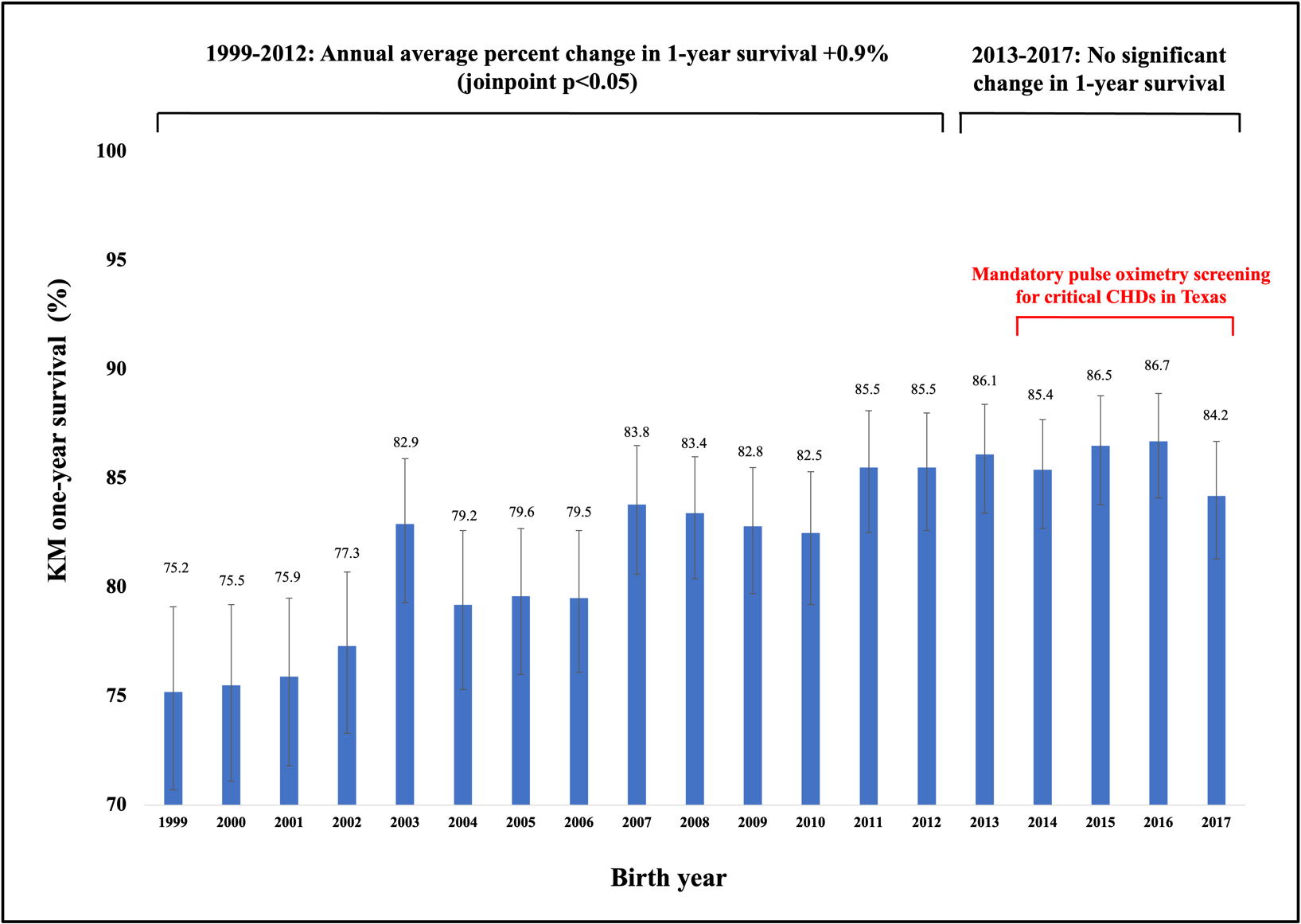


^a^CHD: Congenital heart defects; KM: Kaplan-Meier. Error bars correspond to 95% KM confidence limits. Percentage infant mortality (%) by year is provided above each confidence bar.

**SUPPLEMENTAL FIGURE 3. Survival to Age 1 Year by Birth Year Among Infants with CHD, Texas, 1999-2017 Births**

**
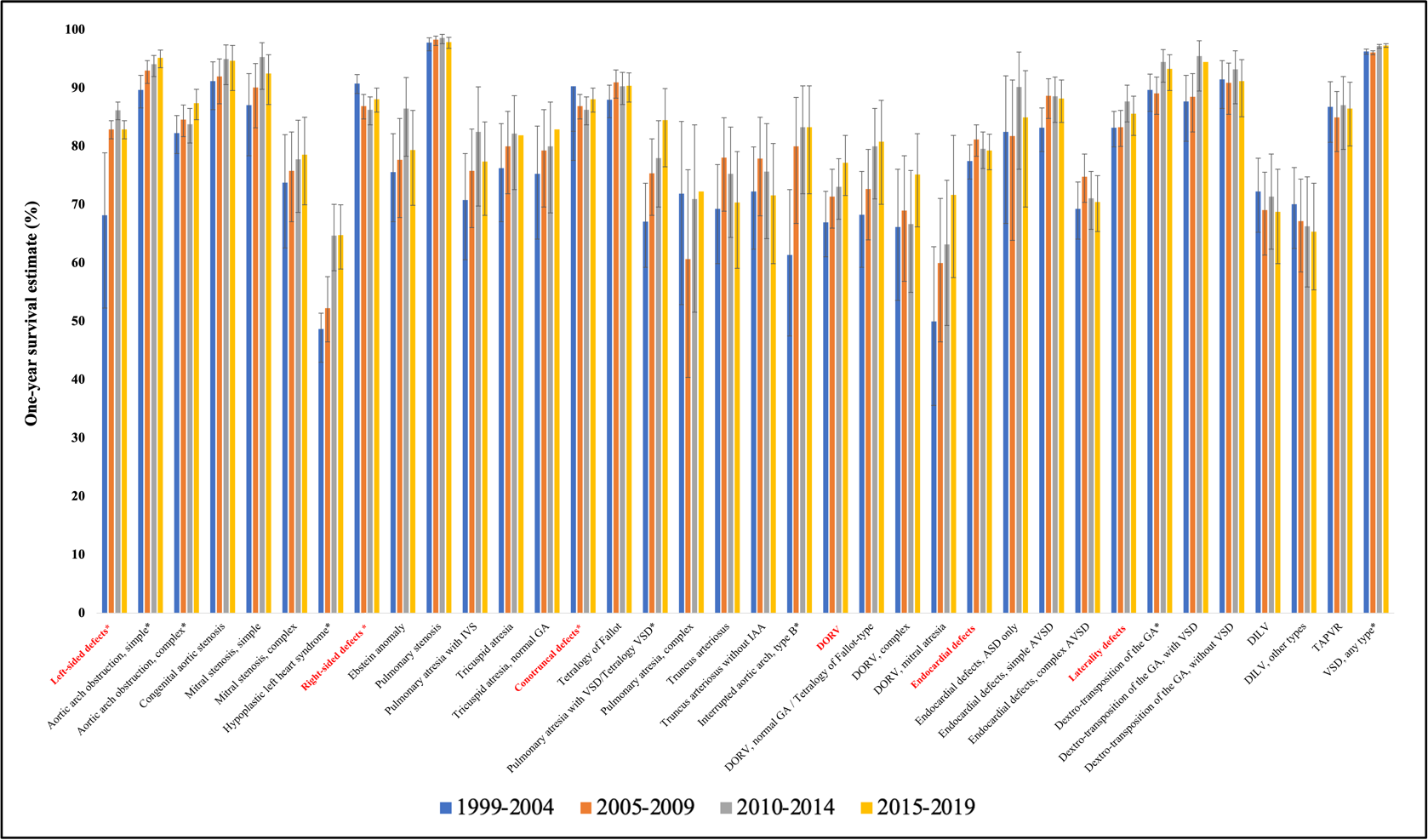
**

**SUPPLEMENTAL FIGURE 4. Survival to Age 1 Year by Maternal Race/Ethnicity Among Infants with CHD, Texas, 1999-2017 Births**

**
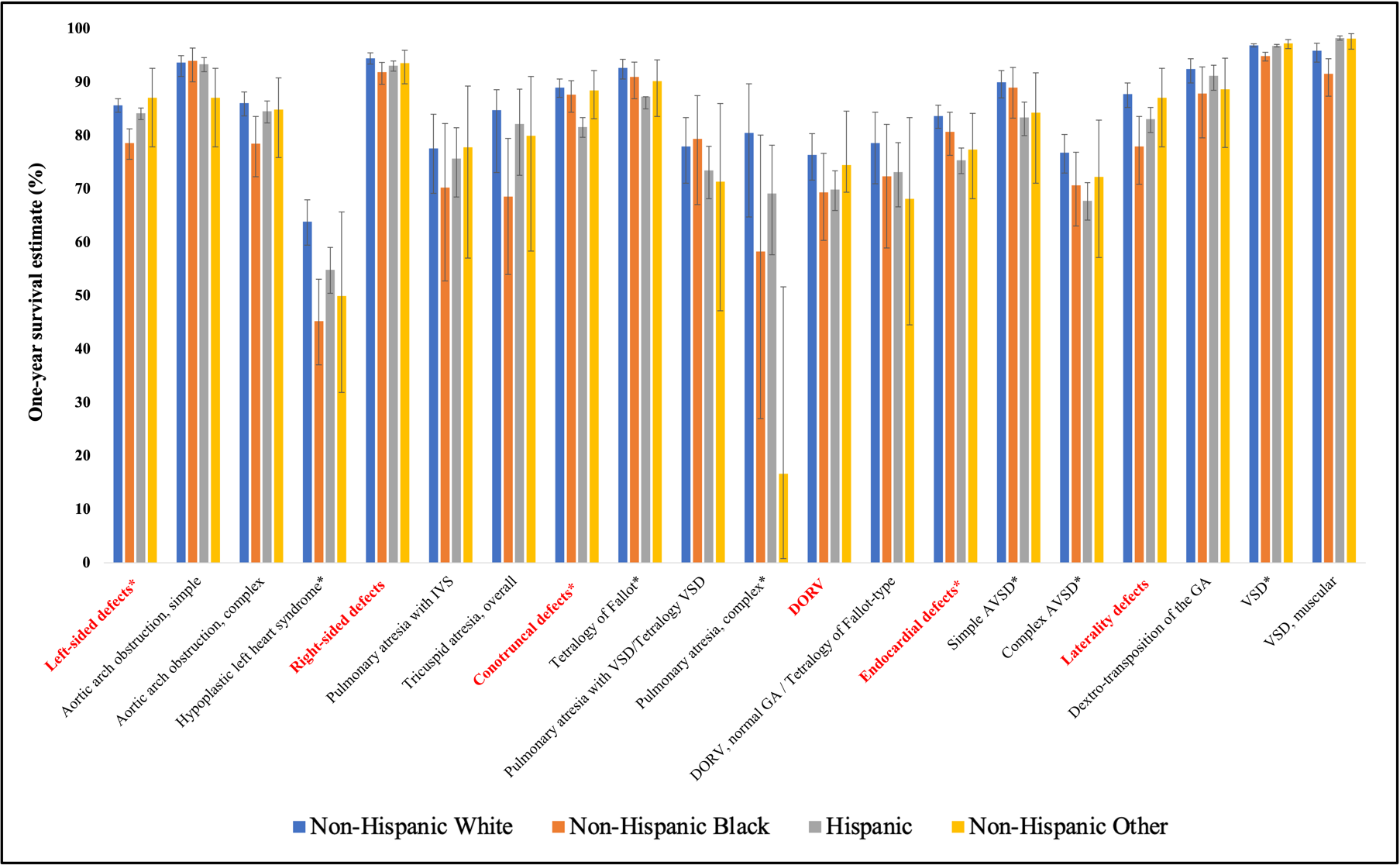
**

**SUPPLEMENTAL FIGURE 5. Survival Estimates to Age 1 Year by Infant Sex Among Infants with CHD, Texas, 1999-2017 Births^a^**


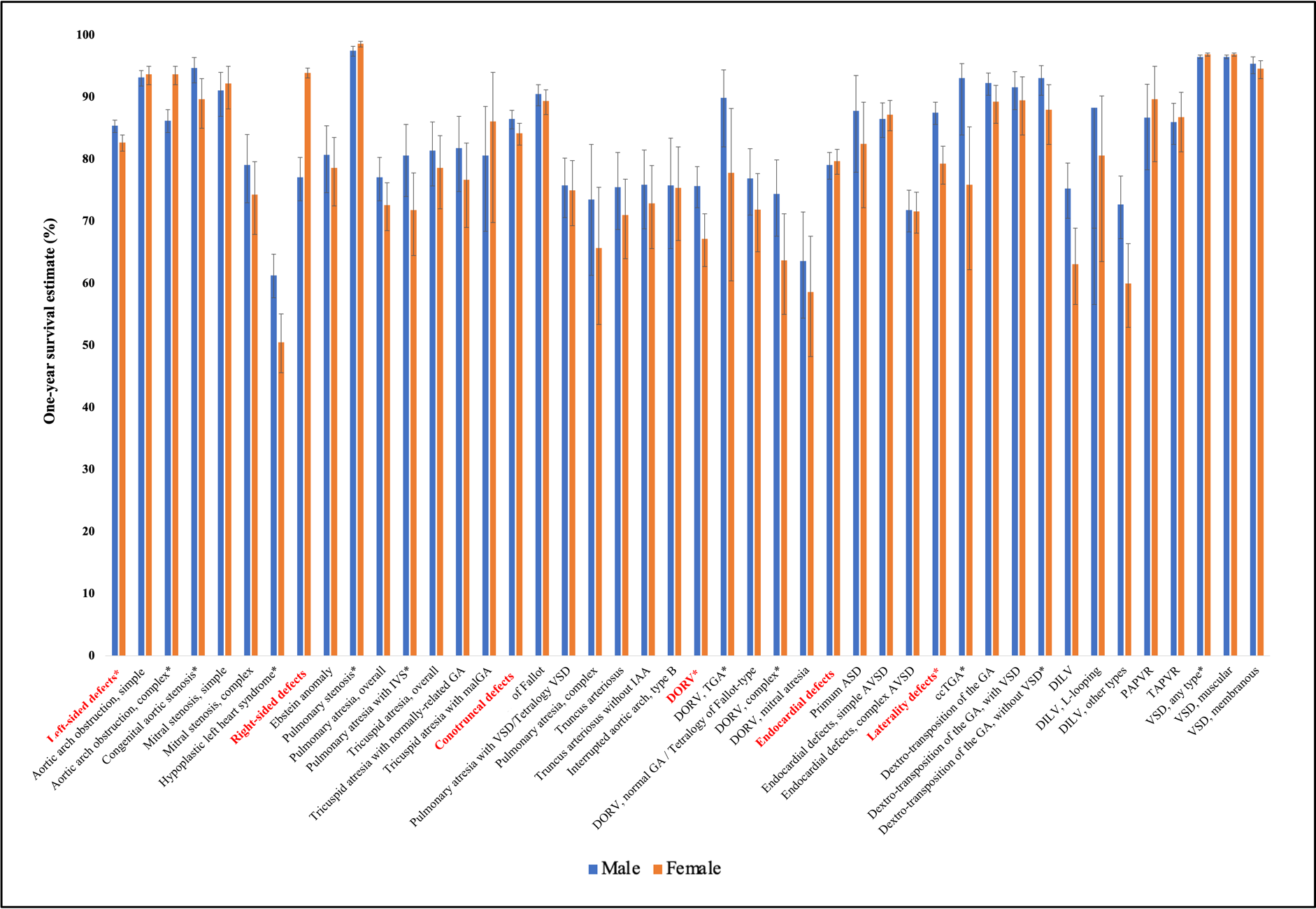


AV: Atrioventricular; CHD: Congenital heart defects; D: Day; dTGA: Dextro-transposition of the great arteries; DILV: Double inlet left ventricle; DORV: Double outlet left ventricle; GA: Great arteries; IAA: Interrupted aortic arch; IQR: Interquartile range; IVS: Intact ventricular septum; PV: Pulmonary venous; Synd.: Syndrome; TGA: Transposition of the great arteries; TOF: Tetralogy of Fallot; Truncus: Truncus arteriosus; VSD: Ventricular septal defect.

^a^An asterisk next to the CHD corresponds to a log-rank p<0.05. Survival estimates for CHDs with <5 deaths within a given category were not computed. Error bars correspond to 95% confidence limits.

**SUPPLEMENTAL FIGURE 6. Kaplan-Meier Survival Estimates Among Infants with CHD by Gestational Age, Texas, 1999-2017 Births**

**
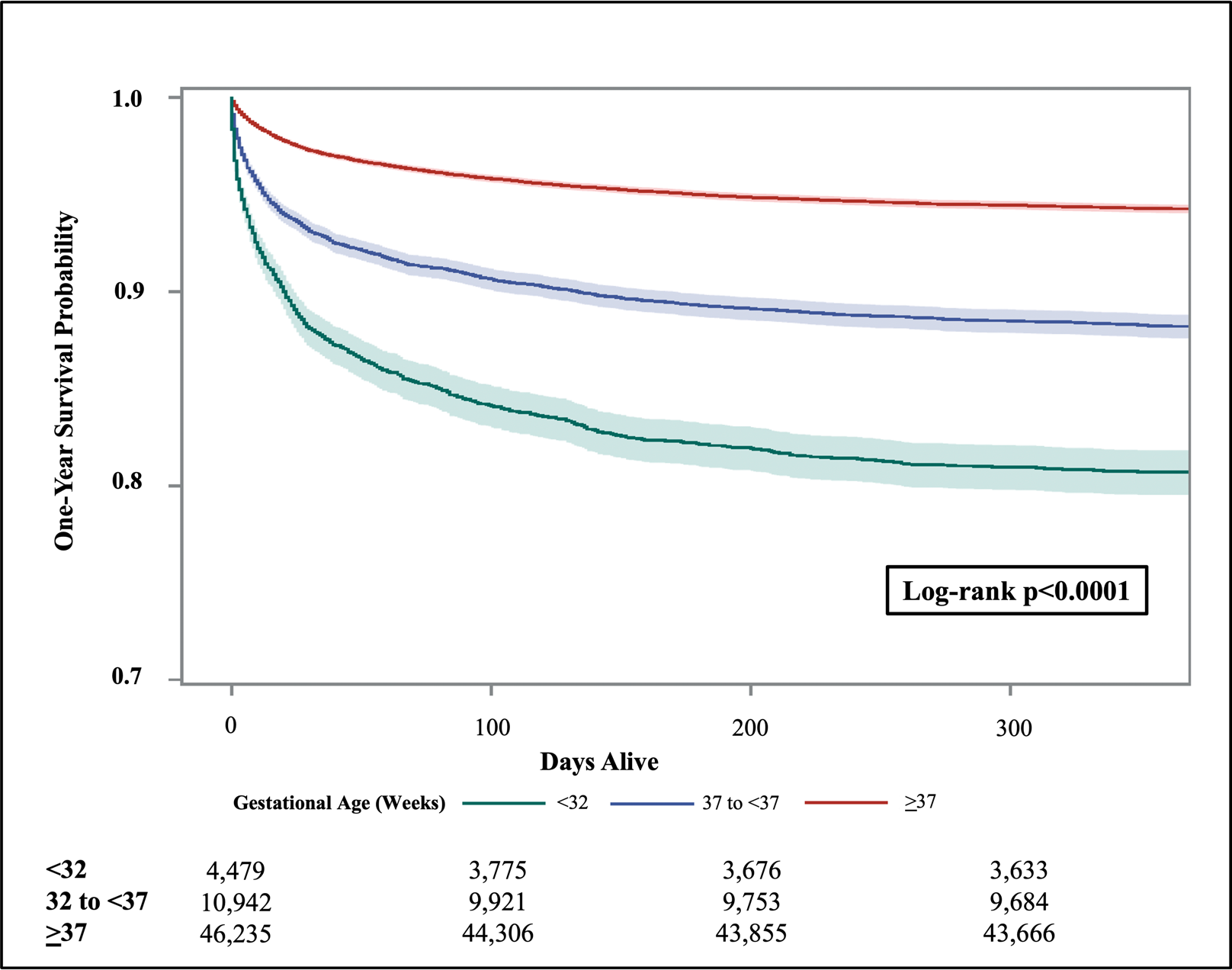
**

**SUPPLEMENTAL FIGURE 7. Survival to Age 1 Year by Gestational Age Among Infants with CHD, Texas, 1999-2017 Births^a^**

**
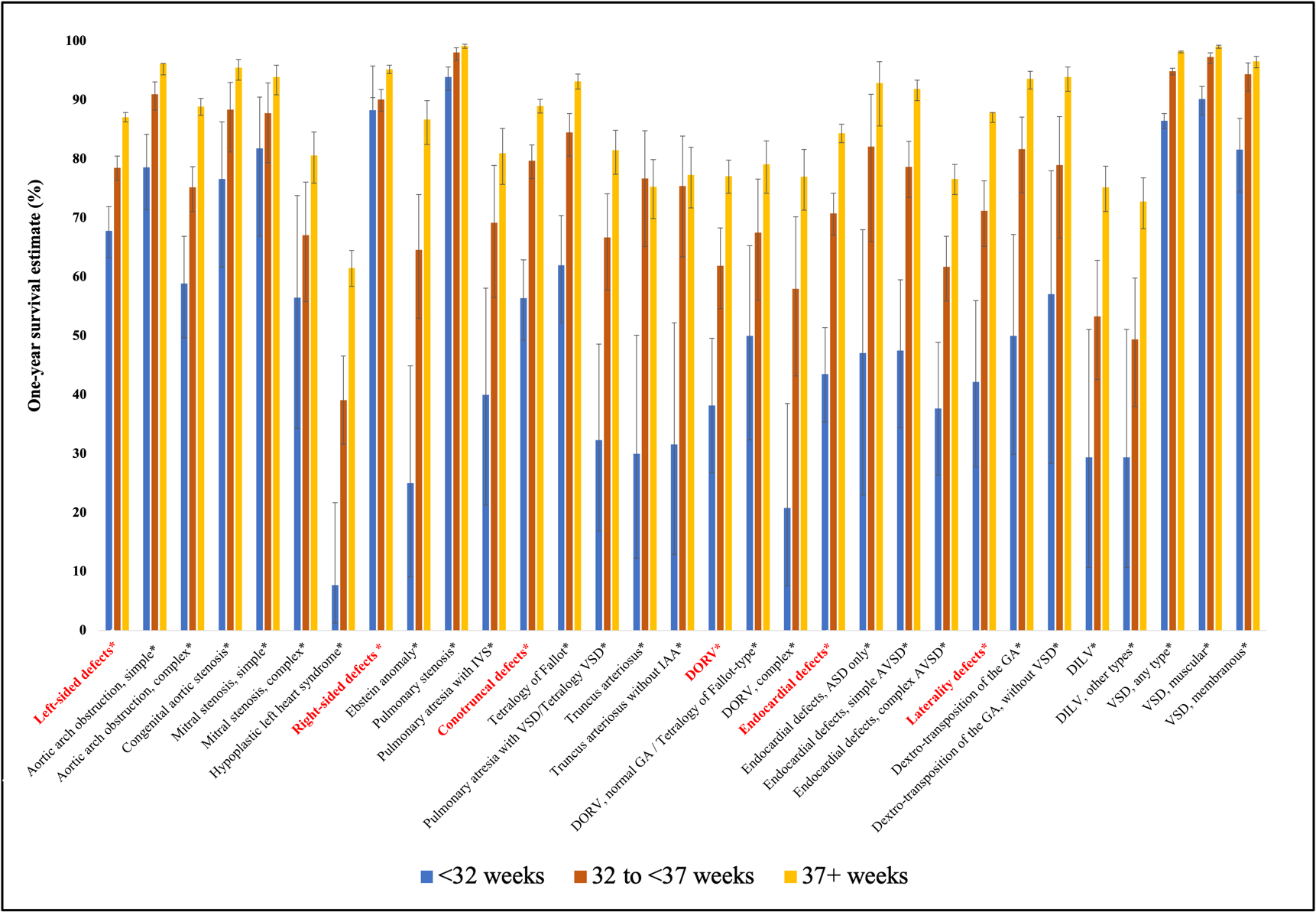
**

AV: Atrioventricular; CHD: Congenital heart defects; D: Day; dTGA: Dextro-transposition of the great arteries; DILV: Double inlet left ventricle; DORV: Double outlet left ventricle; GA: Great arteries; IAA: Interrupted aortic arch; IQR: Interquartile range; IVS: Intact ventricular septum; PV: Pulmonary venous; Synd.: Syndrome; TGA: Transposition of the great arteries; TOF: Tetralogy of Fallot; Truncus: Truncus arteriosus; VSD: Ventricular septal defect.

^a^An asterisk next to the CHD corresponds to a log-rank p<0.05. Survival estimates for CHDs with <5 deaths within a given category were not computed. Error bars correspond to 95% confidence limits. Infants were evaluated by categorized gestational age at birth.

**SUPPLEMENTAL FIGURE 8. Kaplan-Meier Survival Estimates Among Infants with CHD by Low Birthweight (LBW), Texas, 1999-2017 Births**

**
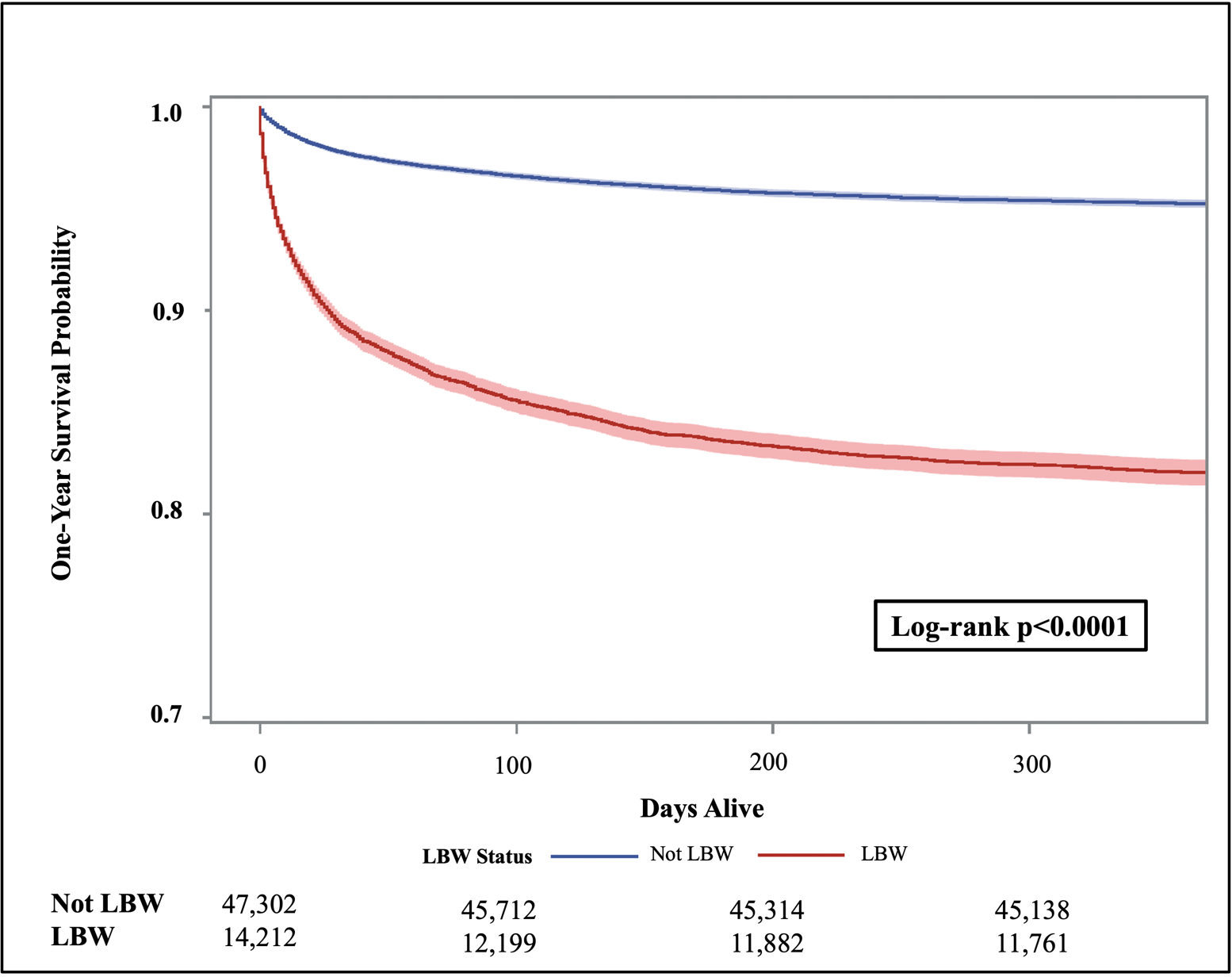
**

**SUPPLEMENTAL FIGURE 9. Survival to Age 1 Year by Low Birthweight Among Infants with CHD, 1999-2017 Births^a^**

**
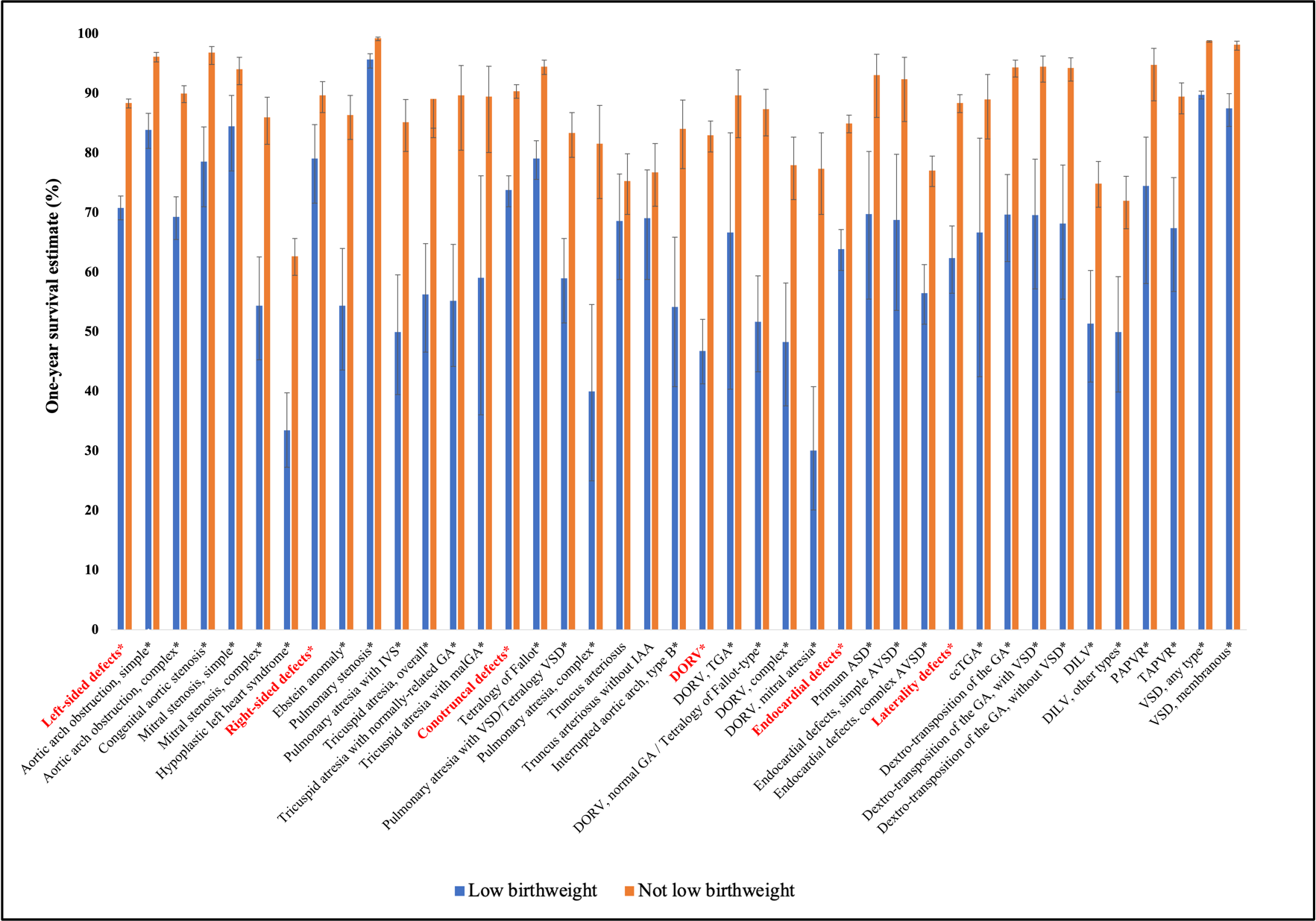
**

AV: Atrioventricular; CHD: Congenital heart defects; D: Day; dTGA: Dextro-transposition of the great arteries; DILV: Double inlet left ventricle; DORV: Double outlet left ventricle; GA: Great arteries; IAA: Interrupted aortic arch; IQR: Interquartile range; IVS: Intact ventricular septum; PV: Pulmonary venous; Synd.: Syndrome; TGA: Transposition of the great arteries; TOF: Tetralogy of Fallot; Truncus: Truncus arteriosus; VSD: Ventricular septal defect.

^a^An asterisk next to the CHD corresponds to a log-rank p<0.05. Survival estimates for CHDs with <5 deaths within a given category were not computed. Error bars correspond to 95% confidence limits.

**SUPPLEMENTAL FIGURE 10. Infantile Survival Estimates by Presence of Extracardiac Defect Among Nonsyndromic Infants with CHD, Texas, 1999-2017 Births^a^**

**^
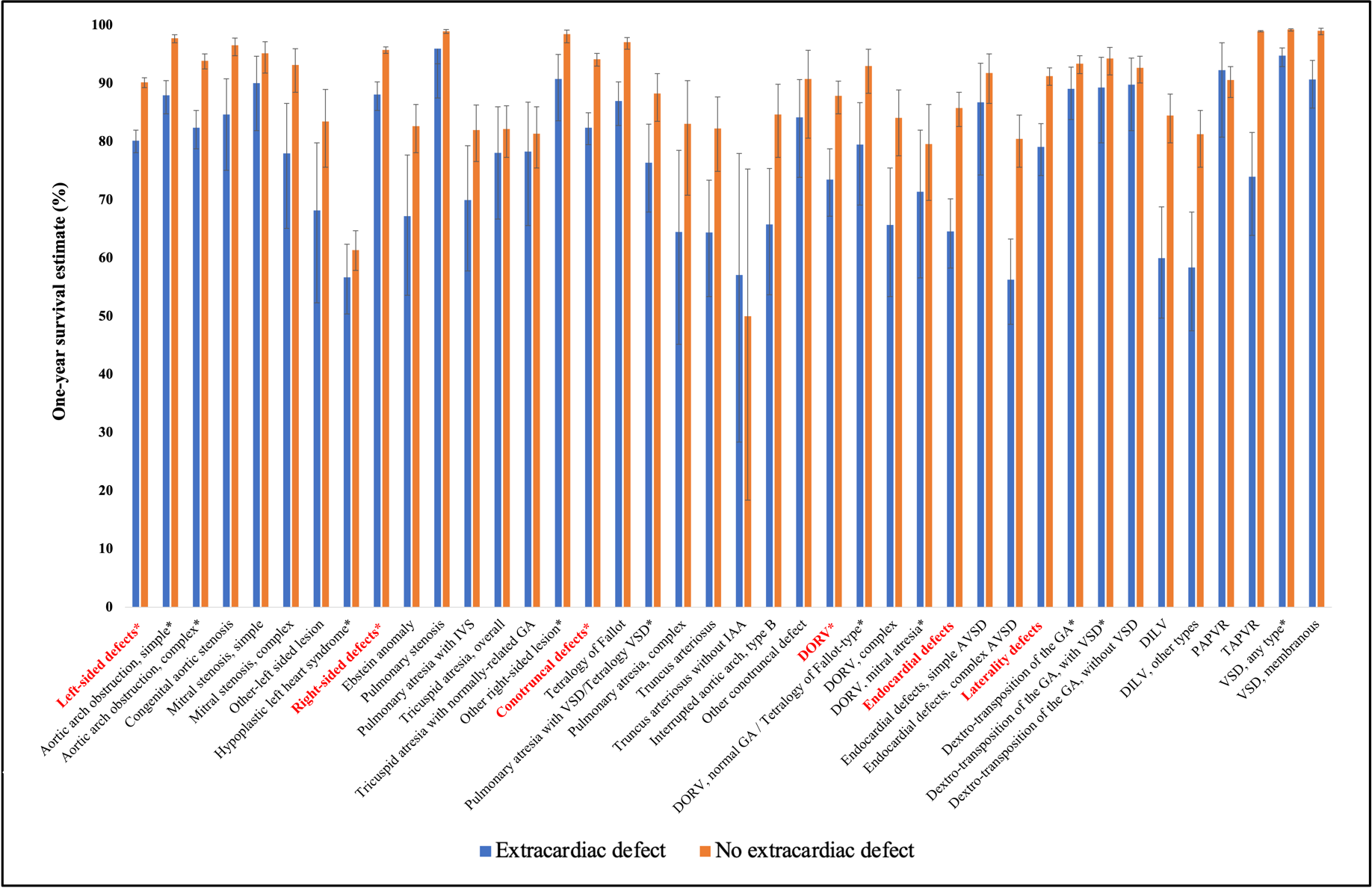
^**

AV: Atrioventricular; CHD: Congenital heart defects; D: Day; dTGA: Dextro-transposition of the great arteries; DILV: Double inlet left ventricle; DORV: Double outlet left ventricle; GA: Great arteries; IAA: Interrupted aortic arch; IQR: Interquartile range; IVS: Intact ventricular septum; PV: Pulmonary venous; Synd.: Syndrome; TGA: Transposition of the great arteries; TOF: Tetralogy of Fallot; Truncus: Truncus arteriosus; VSD: Ventricular septal defect.

^a^An asterisk next to the CHD corresponds to a log-rank p<0.05. Survival estimates for CHDs with <5 deaths within a given category were not computed. Error bars correspond to 95% confidence limits.

**SUPPLEMENTAL FIGURE 11. Kaplan-Meier Survival Estimates Among Infants with CHD by Genetic and Extracardiac Defect (ECD) Status, Texas, 1999-2017 Births**

**
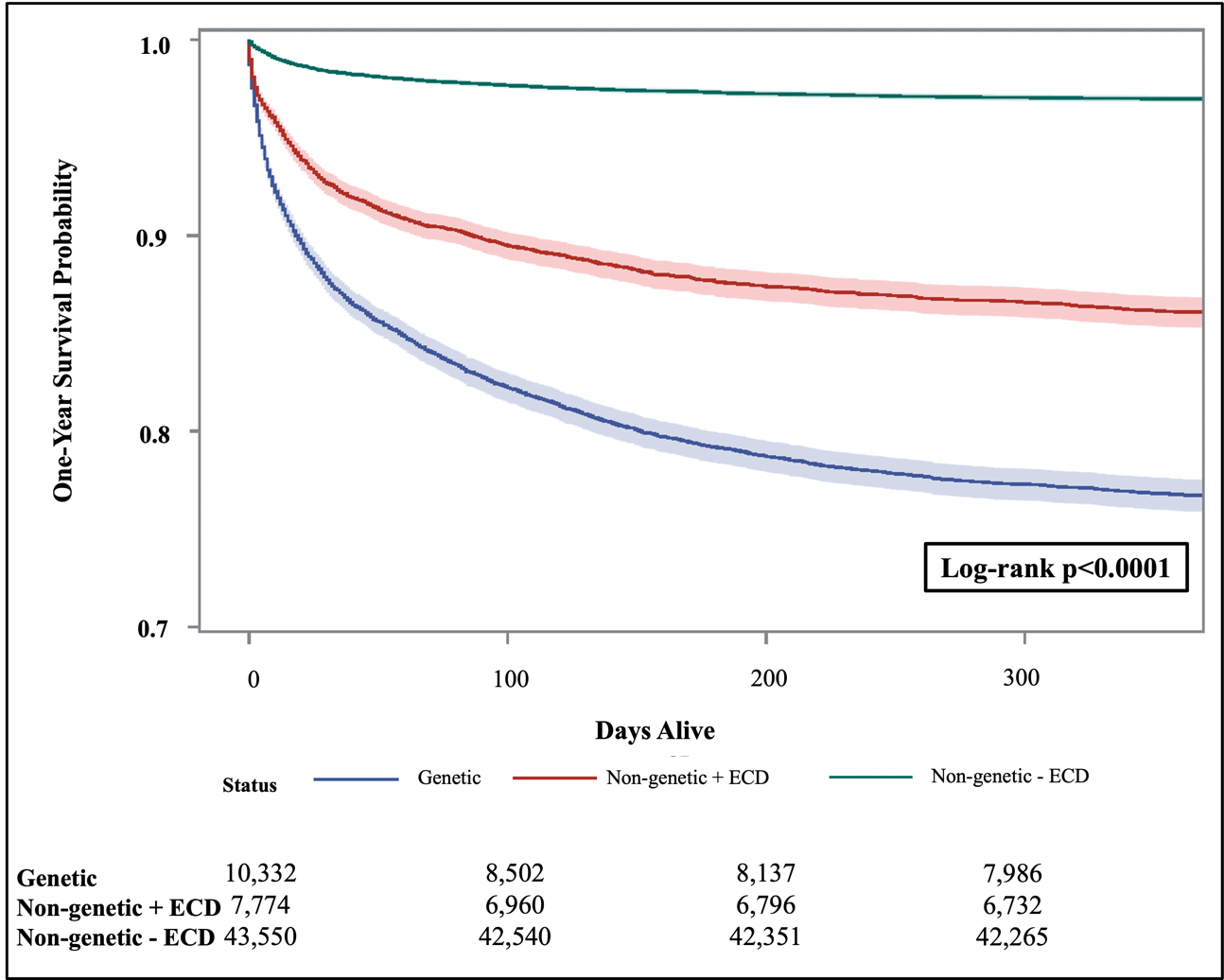
**
